# TRPV4-induced Müller cell gliosis and TNF-α elevation-mediated retinal ganglion cell apoptosis in glaucomatous rats via JAK2/STAT3/NF-κB pathway

**DOI:** 10.1186/s12974-021-02315-8

**Published:** 2021-11-17

**Authors:** Qian Li, Yun Cheng, Shenghai Zhang, Xinghuai Sun, Jihong Wu

**Affiliations:** 1 Eye Institute, Eye and ENT Hospital, College of Medicine, Fudan University, Shanghai, China; 2 Shanghai Key Laboratory of Visual Impairment and Restoration, Science and Technology Commission of Shanghai Municipality, Shanghai, China; 3 NHC Key Laboratory of Myopia, Fudan University, Shanghai, China; 4 Key Laboratory of Myopia, Chinese Academy of Medical Sciences, National Health Commission, #83 Fenyang Road, 200031 Shanghai, China

**Keywords:** TRPV4, Retinal Müller cell, TNF-α, Apoptosis, Glaucoma

## Abstract

**Background:**

Glaucoma, the leading cause of irreversible blindness worldwide, is a type of retinal disease characterized by the selective death of retinal ganglion cells (RGCs). However, the pathogenesis of glaucoma has not been fully elucidated. Transient receptor potential vanilloid 4 (TRPV4) is a pressure-sensitive and calcium-permeable cation channel. TRPV4 is widely distributed in the retina and its sustained activation leads to RGC death; indicating that TRPV4 may be a possible target for glaucoma treatment. Here, we investigated the effects of TRPV4 on RGC apoptosis in a rat model of chronic ocular hypertension (COH), then examined the mechanism underlying these effects.

**Methods:**

The COH model was established by injection of micro-magnetic beads into the anterior chamber of adult male rats. The expression levels of TRPV4, glial fibrillary acidic protein, and inflammatory factors were assessed by immunohistochemistry and immunoblotting. RGC apoptosis and visual dysfunction were evaluated by TUNEL assay and photopic negative response. Functional expression of TRPV4 was examined by electrophysiology and calcium imaging. Real-time polymerase chain reaction and immunoblotting were employed to investigate the molecular mechanism underlying the effects of TRPV4 on tumor necrosis factor-α (TNF-α) release.

**Results:**

We found that TRPV4 played an essential role in glaucoma, such that high levels of TRPV4 expression were associated with elevated intraocular pressure. Furthermore, TRPV4 activation was involved in glaucoma-induced RGC apoptosis and RGC-related reductions in visual function. Mechanistic investigation demonstrated that TRPV4 activation led to enhanced Müller cell gliosis and TNF-α release via the JAK2/STAT3/NF-kB pathway, while TRPV4 inhibition could reverse these effects. Finally, TRPV4 activation could lead to elevated expression of TNF receptor 1 in RGCs, while inhibition of TNF-α could reduce TRPV4-mediated RGC apoptosis.

**Conclusions:**

TRPV4 activation induces Müller cell gliosis and TNF-α elevation via the JAK2/STAT3/NF-κB pathway, which may exacerbate RGC apoptosis in glaucoma; these results suggest that TRPV4 can serve as a therapeutic target in glaucoma treatment.

**Supplementary Information:**

The online version contains supplementary material available at 10.1186/s12974-021-02315-8.

## Background

Glaucoma is the leading cause of irreversible blindness worldwide. Elevated intraocular pressure (IOP) is one of the risk factors for glaucoma, while retinal ganglion cell (RGC) apoptosis is the core etiology of glaucoma [1]. Glaucoma is a retinal neurodegenerative disease with complex pathogenesis. Considerable efforts have been made to identify the molecular basis of RGC death; proposed mechanisms include intrinsic and extrinsic apoptotic signal activation, mitochondrial dysfunction, axonal transport failure, neurotrophic factor deprivation, and excitotoxic damage [2–5]. However, the mechanism underlying RGC death has not been fully elucidated.

Transient receptor potential vanilloid 4 (TRPV4) is a nonselective cation channel with wide distribution in diverse cells (e.g., neurons, glia, and endothelial cells) [6–9]. TRPV4 is sensitive to multiple stimuli and participates in many physiological processes [10–13]. Intracellular calcium overload, caused by TRPV4 hyperactivation, induces neuronal damage in various neurological diseases [14–16]. Previous studies have shown that TRPV4 is expressed in RGC somata, axons, and optic nerve heads, as well as Müller cells, in mouse retina [17, 18], Notably, TRPV4 activation can lead to an increased intracellular calcium ion concentration [17–19]. Continuous channel activation can induce Müller cell gliosis in mouse retina, as well as apoptosis in cultured mouse RGCs and adult porcine RGCs [18, 20]. In glaucoma, optic disc cupping may lead to RGC axon stretching [21, 22]. Therefore, TRPV4, a type of mechanosensitive channel, might be activated by pathologically elevated IOP [23, 24]. However, TRPV4 expression and the specific mechanisms underlying TRPV4-mediated RGC injury in glaucoma have not been fully elucidated.

Müller cells, a major type of glial cells in the retina, have crucial roles in the regulation and maintenance of RGC function [25, 26]. In glaucomatous conditions, Müller cells undergo reactivation (gliosis) [26, 27], which is characterized by the upregulation of glial cytoskeletal proteins, glial fibrillary acidic protein (GFAP), and vimentin [28–31]. There is considerable evidence that Müller cell gliosis may be involved in retinal neurodegeneration and the induction of RGC apoptosis through the release of inflammatory cytokines [26]. In the central nervous system, TRPV4 activation may enhance inflammation and induce cytotoxicity, but it remains unknown whether these effects are involved in glaucoma-related RGC damage.

In the present study, we first show that TRPV4 expression is significantly upregulated in the retinas of rats with chronic ocular hypertension (COH). Furthermore, this study showed that TRPV4 activation could enhance Müller cell gliosis and RGC apoptosis, thereby reducing visual function; pre-inhibition of TRPV4 could alleviate these effects. In addition, TRPV4 activation could enhance the release of inflammatory cytokines (e.g., tumor necrosis factor-α [TNF-α]) through JAK2/STAT3/NF-kB signaling pathways in Müller cells and elevated expression of TNF receptor 1 in RGCs; this process is involved in RGC apoptosis during glaucoma. Overall, these results suggest that TRPV4 activation promotes RGC apoptosis by the enhancement of Müller cell gliosis and release of inflammatory cytokines.

## Methods

### Animals

All experimental animal procedures were performed in accordance with the National Institutes of Health guidelines for the Care and Use of Laboratory Animals, as well as the guidelines of Fudan University for the ethical use of animals. Wistar rats (weighing 180–200 g) were purchased from SLAC Laboratory Animal Co., Ltd. (Shanghai, China). Sox2-Cre mice (B6.Cg-Tg(Sox2-cre)1Amc/J) were crossed with Rosa26 mice (B6. Cg-Gt(ROSA)26Sor tm14(CAG-tdTomato)Hze/J) obtained from Jackson Laboratory Animal Co., Ltd. (USA) to create TdTomato-labeled Müller-transgenic mice.

### Rat model of COH

**Fig. 1:**
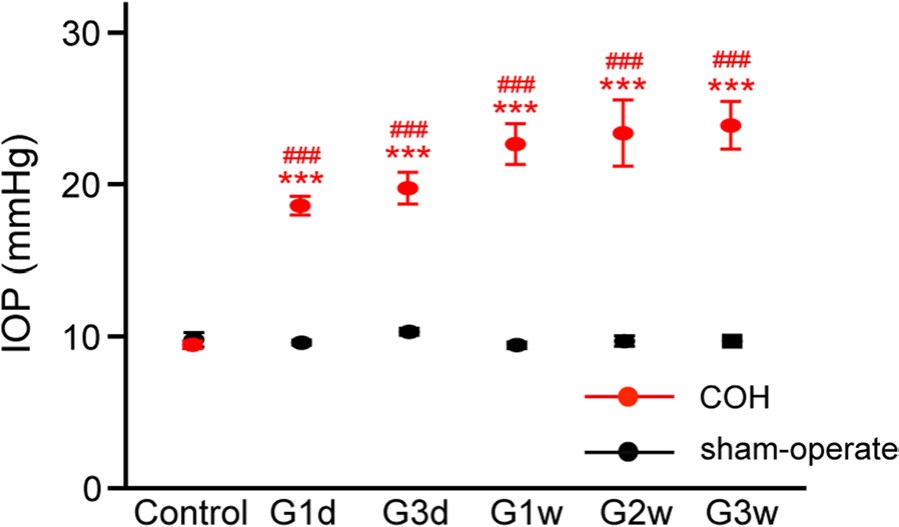
Rat chronic ocular hypertension (COH) model. Mean intraocular pressure (IOP) before (control) and after (≥ 1 day) a single unilateral injection of micro-magnetic beads (8 µl) in rat retinas, showing microbead-induced elevations in IOP (COH). *n* = 9–47. ***p < 0.001 vs sham-operated treatment at the same timepoint and ^###^*p* < 0.001 vs control

COH modeling was performed as in our previous studies [32]. Briefly, rats were anesthetized deeply with a mixture of ketamine (25 mg/kg, im) and xylazine (10 mg/kg, im); eyes were locally anesthetized via topical application of 0.4% oxybuprocaine hydrochloride eyedrops (Benoxil, Santen Pharmaceutical Co. Ltd., Osaka, Japan). Micro-magnetic beads (8 μl, BioMag® Superparamagnetic Iron Oxide, Bangs Laboratories, Inc., Fisher, IN, USA) were injected into the anterior chamber of the right eye. Sham injection (0.9% saline) was performed in a conventional manner in the contralateral eye (left eye); this served as the sham-operated group. IOP was measured using a handheld digital tonometer (Tonolab, TioLat, Finland); measurements were performed in the morning to avoid possible circadian differences. The IOPs of both eyes were recorded before surgery (control); they were also recorded at 1 day, 3 days, 1 week, 2 weeks, and 3 weeks after surgery (Fig. 1).

### Intravitreal injection

Intravitreal injections were performed as in our previous studies [33]. The TRPV4 agonist, GSK1016790A (GSK101) (1 µM or 10 µM); TRPV4 antagonist, HC-067047 (HC-067) (10 µM); or inhibitor of soluble TNF-α, R7050 (1 µM), were suspended in 2 μl of 0.9% saline and injected into the vitreous space at a post-limbal location. HC-067 and R7050 were pre-injected 1–2 days before GSK101 injection; samples were collected for analysis at 1 week after injection of GSK101. HC-067 was pre-injected 2 days before initiation of COH modeling; samples were collected for analysis at 2 weeks after COH model establishment. Eyes that received an injection of saline (2 μl) alone in the same manner served as negative controls.

### Immunoblotting

Immunoblotting was performed as previously described, using the Wes Simple Western system (ProteinSimple, San Jose, CA, USA) [33]. For whole-cell protein extraction, retinas were rapidly collected, then homogenized in RIPA lysis buffer that had been supplemented with protease and phosphatase inhibitor cocktails (Roche Applied Science, Mannheim, Germany). Protein concentrations of whole-cell extracts were measured using a standard bicinchoninic acid assay kit (Pierce Biotechnology, Rockford, IL, USA), then analyzed using a Wes Simple Western instrument (ProteinSimple), in accordance with the manufacturer’s instructions. Protein samples were mixed with fluorescent 5X master mix (ProteinSimple), then heated at 95 °C for 5 min. The total quantity of protein used for immunoblotting was 40 ng per sample. The following components were loaded into the Wes plate (Wes 12–230 kDa or 2–40 kDa Pre-filled Plates with Split Buffer, ProteinSimple): boiled samples, biotinylated protein ladder, blocking buffer, primary antibodies, ProteinSimple horseradish peroxidase-conjugated anti-rabbit or anti-mouse secondary antibodies, luminol-peroxide, and wash buffer. Plates and capillary cartridges were loaded into the Wes instrument; protein separation, antibody incubation, and imaging were performed using default parameters. Compass software (ProteinSimple) was used to acquire the data, then perform image reconstruction and examine chemiluminescence signal intensity. Protein and phosphorylation levels were expressed as the area of peak chemiluminescence intensity. The following primary antibodies were used: anti-TRPV4 (cat. no. LS-C94498, 1:20, Labome), anti-GFAP (cat. no. ab7260, 1:500, Abcam, Cambridge, MA, USA), anti-TNF-a (cat. no. PB0270, 1:10, Boster or cat. no. ab6671, 1:10, Abcam), anti-STAT3 (cat. no. ab68153, 1:500, Abcam), anti-phosphorylated STAT3 (cat. no. ab76315, 1:50, Abcam), anti-JAK2 (cat. no. ab32101, 1:50, Abcam), anti-phosphorylated JAK2 (cat. no. mAb3776, 1:10, Cell Signaling Technology, Danvers, MA, USA), anti-NLRP3 (cat. no. ab263899, 1:100, Abcam), anti-caspase 1 (cat. no. AF5418, 1:10, Affinity), anti-TNF receptor 1 (cat. no. ab90463, 1:50, Abcam), and anti-GAPDH (cat. no. D16H11, 1:200, Cell Signaling Technology).

Immunoblotting of nuclear protein extracts was performed as previously described [34]. For nuclear protein extraction, the Nucleus-Cytosol Extraction kit (Applygen Technologies, Inc., Beijing, China) was used, in accordance with the manufacturer’s instructions. The extracted protein samples were separated on an 10% SDS-PAGE gel and electrotransferred to PVDF membranes (Immobilon-P, Millipore, Billerica, MA, USA). The following primary antibodies were used: anti-NF-κB p65 (cat. no. 10745-1-AP, 1:2000, Proteintech) and anti-lamin B receptor (cat. no. ab32535, 1:500, Abcam). The membranes were incubated with donkey anti-mouse, anti-rabbit, or anti-goat IgG HRP (Jackson ImmunoResearch Labs) for 1.5 h at room temperature; they were then incubated with enhanced chemifluorescence reagent (Pierce Biotechnology). The blots were imaged with a digital imager (FluorChem E System, ProteinSimple) and protein bands were quantitatively analyzed with Alpha View software (Cell Biosciences, Inc.).

### Immunohistochemistry

Immunohistochemistry was performed as described in previous studies [32, 34]. Briefly, Müller cells were cultured on cover slips, then fixed with 4% paraformaldehyde for 20 min. For analysis of rat tissue, retinas were fixed with 4% paraformaldehyde for 2 h and dehydrated with graded sucrose solutions at 4 °C, then vertically sectioned at a thickness of 10 μm (Leica, Nussloch, Germany). After the cultured cells or retinal sections had been washed in PBS, they were blocked for 1.5 h in 10% donkey serum, 3% BSA, and 0.1% Triton X-100. Subsequently, they were incubated with the following primary antibodies at 4 °C overnight: anti-TRPV4 (cat. no. ACC-034, 1:200, Alomone Labs), anti-glutamine synthetase (GS, cat. no. GTX109121, 1:400, GeneTex), anti-GFAP (cat. no. bs-0199R, 1:500, Bioss), and anti-NF-κB p65 (cat. no.sc-372, 1:200, Santa Cruz). As negative controls, TRPV4 and GFAP antibodies were pre-adsorbed with TRPV4 blocking peptide (Alomone Labs) and GFAP blocking peptide (Bioss), respectively. Binding sites of the primary antibody were visualized by incubation with Alexa Fluor 488-conjugated goat anti-mouse IgG (1:500 dilution) and Alexa Fluor 555-conjugated donkey anti-rabbit IgG (1:500 dilution, both secondary antibodies from Invitrogen-Molecular Probes) for 1 h at room temperature. Sections were sealed under coverslips with anti-fade mounting medium containing 4′,6-diamidino-2-phenylindole (DAPI, Vector Laboratories, Burlingame, CA, USA); immunofluorescence images were visualized with a confocal laser scanning microscope (FluoView 1000, Olympus, Tokyo, Japan).

### Retinal slices and electrophysiological recordings

Rats were deeply anesthetized; their eyes were enucleated quickly, then immersed in ice-cold artificial cerebrospinal fluid (ACSF) containing (in mM): NaCl 125, KCl 3, NaHCO_3_ 26, Na_2_HPO_4_ 1.25, CaCl_2_ 2, MgCl_2_ 1, and glucose 15 (pH 7.4), with 95% O_2_ and 5% CO_2_ bubbled through the solution. Subsequently, retinas were isolated and sliced vertically at a thickness of 200 μm on a Narishige slicer (ST-20-P, Tokyo, Japan). Slices were transferred to a holding chamber, where they were fully submerged in oxygenated ACSF solution and maintained at room temperature (24–25 °C) for 30 min before recording. Whole-cell voltage and current-clamp recordings were performed using standard techniques, as described previously [32]. Individual slices were transferred to a chamber that was continuously superfused with oxygenated ACSF at a rate of 1–2 ml/min at room temperature. RGCs were identified by their locations and morphologies, then further identified by intracellular injection of Alexa Fluor 488.

Cells were detected with a charge-coupled device camera and displayed on a monitor. Patch pipettes were made by pulling BF150-86-10 glass (Sutter Instrument Co., Novato, CA, USA) onto a P-97 Flaming/Brown micro-pipette puller (Sutter Instrument Co.), then fire-polished (Model MF-830, Narishige, Japan) before recording. Pipette resistance was typically 4–8 MΩ after it had been filled with internal solution. The internal solution consisted of (in mM): potassium D-gluconate 120, ethylene glycol-bis (β-aminoethyl ether) N,N,N′,N′-tetraacetic acid (EGTA) 1, 4-(2-hydroxyethyl) piperazine-1-ethanesulfonic acid (HEPES) 10, ATP·Mg 4, GTP·Na 0.3, phosphocreatine 10, CaCl_2_ 0.1, MgCl_2_ 1, and Alexa Fluor 488 (pH 7.2), adjusted with KOH, 280–290 mOsm/L. Whole-cell membrane potentials were recorded from Müller cells using a patch amplifier (Axopatch 700B; Molecular Devices, Foster City, CA, USA) with a Digidata 1440A data acquisition board and pClamp 10.2 software. Drugs were delivered by a gravity-driven superfusion system for at least 5 min before assessment of their effects.

### Calcium imaging

Müller cells were seeded on glass coverslips for 24 h, loaded with Fura-2AM (4 μg/ml, Thermo, Waltham, MA, USA) for 30 min, and washed with the bath solution containing (in mM): NaCl 125, KCl 3, NaHCO_3_ 26, Na_2_HPO_4_ 1.25, CaCl_2_ 2, MgCl_2_ 1, and glucose 15 (pH 7.4) for 5–20 min. Excitation was provided via sequential exposure to 340 and 380 nm wavelengths delivered by LAMBDA 10–3 (Sutter Instrument Co.). The images were captured with Cool SNAP HQ2 (Photometrics) and processed with MetaFluor software (Axon). The data were collected as emission ratios for 340 and 380 nm excitations.

### Electroretinography (ERG)

ERG was performed as previously described [35]; the results were recorded using an Espion Diagnosys System (Diagnosys LLC, Littleton, MA, USA). After the pupils had been dilated with phenylephrine hydrochloride and tropicamide (0.5%), recording electrodes were placed in the center of the cornea. The reference electrode was placed hypodermically on the central forehead and the grounding electrode was attached to the tail. For assessment of photopic negative response (PhNR), light stimulation was performed at 20 cd seconds per meter squared (cd.s/m^2^) green light–0.5 Hz against a white background of 30 cd.s/m^2^ for 4 ms. The PhNR value refers to the amplitude from baseline to trough. For scotopic ERG analysis, rats were adapted in darkness overnight before recording, and white flashes of 1 cd.s/m^2^ were applied as flash stimuli. The a-wave (first negative peak) and b-wave (first positive peak) amplitudes were measured and recorded.

### Cell apoptosis assay

To detect cell apoptosis, terminal dUTP nick end labeling (TUNEL) assays were performed on whole flat-mounted retinas, using the DeadEnd Fluorometric TUNEL System G3250 kit (Promega, Madison, WI, USA), as described previously [36]. TUNEL signals were visualized with a confocal laser scanning microscope through a 10X objective (FluoView 1000, Olympus). Each retina was mounted with the ganglion cell layer (GCL) upturned; serial deep scanning was performed only in the GCL, based on the DAPI staining results. All TUNEL-positive signals that merged well with DAPI signals in each retina were counted.

### Retrograde labeling and enumeration of RGCs

Five days before killing, rats were deeply anesthetized. Then, 2 μl of 3% of FluoroGold (Sigma-Aldrich, St. Louis, MO, USA) was injected into the superior colliculus on each side, as previously reported [37]; notably, FluoroGold is taken up by RGC axon terminals and bilaterally transported in a retrograde manner to the cell somata. At the time of killing, the rats’ eyeballs were enucleated and directly fixed in 4% paraformaldehyde for 1.5 h at room temperature. The retinas were then carefully dissected and prepared as flatmounts. To quantify the densities of labeled RGCs, each retina was divided into four quadrants. Sixteen microscopic fields of each retina were counted: two from the central region (1.5 mm from the optic disc) and two from the peripheral region retina (3 mm from the optic disc) for each quadrant. RGC densities (cells/mm^2^) were grouped according to retinal eccentricity (central and peripheral) and expressed as means ± standard errors of the mean (means ± SEMs).

### Cultures of primary retinal Müller cells and RGCs

Primary Müller cell cultures were prepared in accordance with established procedures [34]. Briefly, retinas isolated from newborn Wistar rats (postnatal day 5) were digested with 0.25% trypsin for 5 min at 37 °C. Cell suspensions were cultured in Dulbecco’s modified Eagle medium (DMEM/F12; Gibco, Life Technologies, Rockville, MD, USA), supplemented with 10% fetal bovine serum, 100 U/ml penicillin, and 100 μg/ml streptomycin; they were grown in a humidified atmosphere with 5% CO_2_ at 37 °C. Microglia and unattached cells were removed by blowing with a fire-polished Pasteur pipette. Third-generation Müller cells, cultured for up to 21 days, were used for experiments. RGCs were purified and cultured as previously described [38]. Retinal suspensions were incubated in two anti-rat-macrophage panning plates (Millipore; 15 μl in 7.5 ml of 1 mM Tris buffer, pH 9.5 at 4 °C overnight) at 37 °C for 40 min; each plate was shaken at 20-min intervals. The nonadherent cells were transferred to two anti-rat-Thy1.1 panning plates (Abcam; 15 μl in 7.5 ml of 1 mM Tris buffer, pH 9.5 at 4 °C overnight) at 37 °C for 1 h; each plate was shaken at 20-min intervals. The plates were subsequently washed three times with Dulbecco’s PBS and moderately swirled to dislodge nonadherent cells. Each plate was incubated at 37 °C for 2 min with EBSS media containing 0.25% trypsin (Gibco). Immediately after treatment, DMEM (Gibco) with 30% fetal bovine serum (Gibco) was added to each plate to inactivate the trypsin. After cells had been centrifuged at 200 × g for 5 min, they were seeded on glass coverslips that had been coated with 0.01% poly-d-lysine (Sigma-Aldrich). Purified RGCs were maintained in Neurobasal medium (Gibco) containing supplemental factors and grown at 37 °C in a humidified atmosphere containing 5% CO_2_ and 95% air; these cells were used for experiments. Cultured cells were treated with GSK101 (10 μM) for 24 h. For inhibition experiments, the inhibitor HC-067 (10 μM) was added to the medium 30 min before GSK101 treatment.

### Real-time polymerase chain reaction

Total RNA was isolated from cultured Müller cells using RNAiso Plus (Takara Co., Japan). Real-time polymerase chain reaction assays were performed as previously described [34]. Forward and reverse primer sequences were 5′-ACTGAACTTCGGGGTGATCG-3′ and 5'-GCTTGGTTTGCTACGAC-3′ for TNF-α; 5′-GACTTCACCATGGAACCCGT-3′ and 5'-GGAGACTGCCCATTCTCGAC-3′ for IL-1β; 5′-AGCGATGATGCACTGTCAGA-3′ and 5′-TAGCACACTAGGTTTGCCGA-3′ for IL-6; and 5'-CCGCGAGTACAACCTTCTTG-3′ and 5′-CAGTTGGTGACAATGCCGTG-3′ for β-actin, respectively. The thermal cycling conditions were 95 °C for 2 min, followed by 40 cycles of 45 s at 95 °C, 45 s at 58 °C or 60 °C, and 45 s at 72 °C. The amplification reactions were performed using an amplification device (Eppendorf, realplex 4, GER), which yielded a melting curve. Data were analyzed using the 2^−ΔΔct^ calculation method.

### Statistical analysis

All experiments involving cultured cells were performed at least in triplicate using three separate batches of cultures. Data analysis was performed using Clampfit 10.2 (Molecular Devices), SigmaPlot 14.0 (SyStat, San Jose, CA, USA), GraphPad Prism 6.0 (GraphPad Software, Inc, La Jolla, CA, USA), and Igor 4.0 (WaveMetrics, Lake Oswego, OR, USA). Before any statistical analyses, data were evaluated using the Shapiro–Wilk test or Brown–Forsythe test to determine whether they exhibited normality or homogeneity of variance, respectively. If the Shapiro–Wilk test yielded a *p* value of < 0.05, the Friedman repeated-rank test and Wilcoxon signed-rank test were used instead of ordinary one-way ANOVA and paired *t *tests. If the Brown–Forsythe test yielded a *p* value of < 0.05, the Mann–Whitney *U* test, Friedman test, and Kruskal–Wallis test were used instead of *t* tests, repeated-measures one-way ANOVA (RM one-way ANOVA), and ordinary one-way ANOVA, respectively. Data are expressed as means ± SEMs. A value of *p* < 0.05 was considered statistically significant.

## Results

### TRPV4 protein expression is altered in retinas with COH

Rats with COH exhibited significantly a higher mean IOP in glaucomatous eyes from day 1 to week 3 (G1d to G3w, COH; 19.3 ± 1.02 to 23.4 ± 1.55 mmHg, *n* = 9–28), compared with unoperated eyes (control; 9.33 ± 0.24 mmHg, *n* = 47) and sham-operated eyes (9.44 ± 0.30 to 11.13 ± 0.44 mmHg, *n* = 9–28; all *p* < 0.001, Fig. 1).

**Fig. 2:**
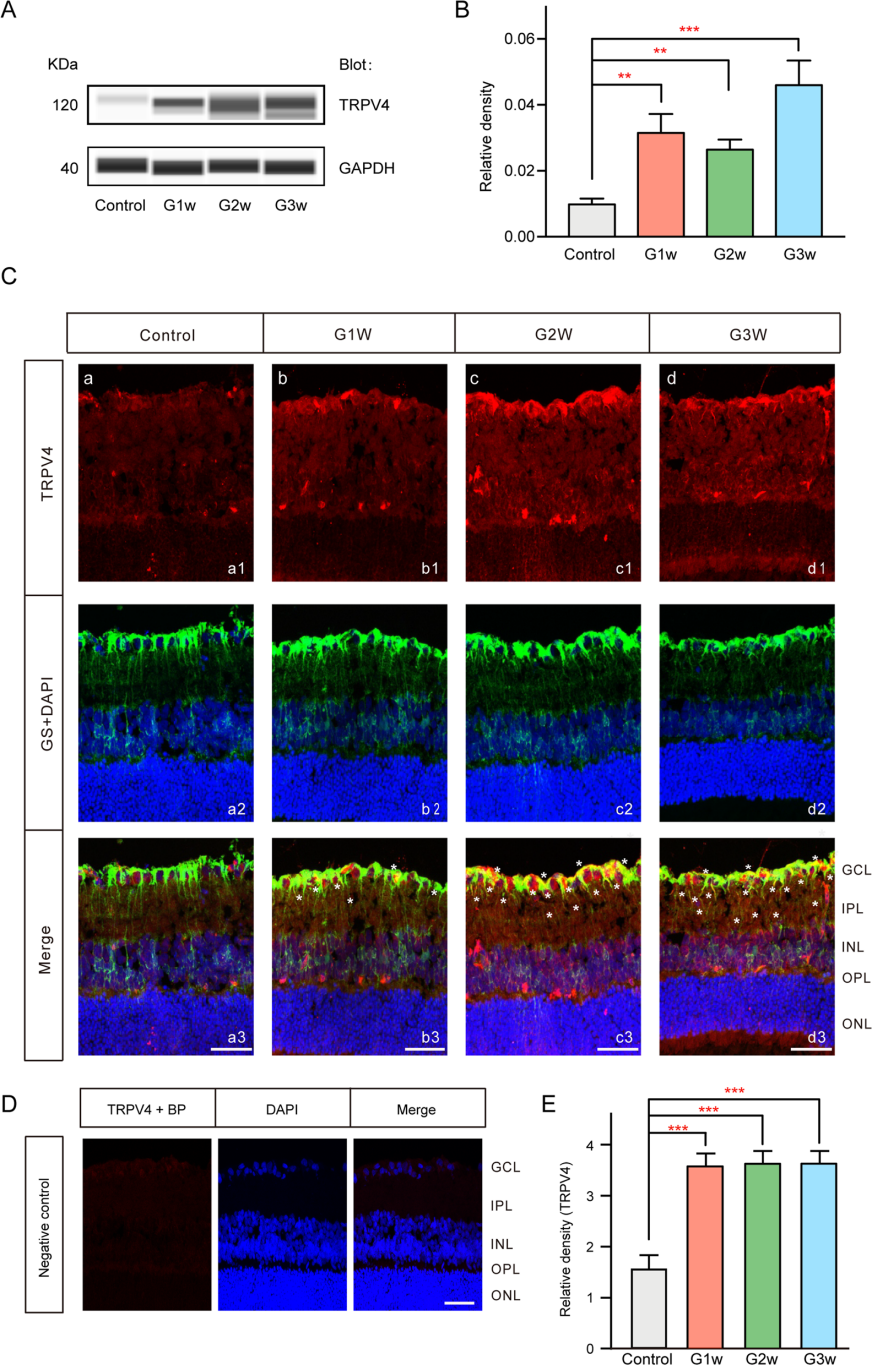
Changes in TRPV4 protein levels in retinas of rats with COH. **A** Representative immunoblots showing changes in TRPV4 protein levels in control and COH retinal extracts at different postoperative times (G1w, G2w, and G3w). **B** Bar chart summarizing mean expression levels of TRPV4 at different postoperative times. Data are presented as means ± standard errors of the mean. *n* = 7, 4, 6, and 5, respectively. **p < 0.01 vs control, ***p < 0.001 vs control. **C** Immunohistochemistry analysis of changes in TRPV4 protein expression patterns in Müller cells from COH retinas. **a1**–**d1**, Immunofluorescence images show TRPV4 protein expression profiles in rat retinal vertical slices collected from control eyes (**a1**) and profiles in COH retinas collected at different postoperative times (G1w, G2w, and G3w) (**b1**–**d1**). **a2**–**d2**, Immunofluorescence images show glutamine synthetase (GS) protein (green) and DAPI (blue) staining profiles in the slices depicted in **a1**–**d1**. **a3**–**d3** depict merged images and the star symbols in **b3**–**d3** represent obvious merge sites. **D** Double immunofluorescence staining showing TRPV4 expression when the TRPV4 antibody was pre-adsorbed with its blocking peptide (BP). Scale bar: 50 µm. *GCL* ganglion cell layer, *IPL* inner plexiform layer, *INL* inner nuclear layer, *OPL* outer plexiform layer, *ONL* outer nuclear layer. **E**, Bar graph summarizing mean density of TRPV4 immunofluorescence in retinas under different conditions. *n* = 4 for all groups. ***p < 0.001

We used immunoblotting to examine whether TRPV4 protein expression changes over time in the retinas of rats with COH. As shown in Fig. 2A, B, the TRPV4 protein level was increased to 320.9% ± 58.1% of control at G1w (*n* = 4, *p* = 0.0046), 269.1% ± 31.2% of control at G2w (*n* = 6, *p* = 0.0067), and 456.3% ± 68.5% of control at G3w (*n* = 5, *p* < 0.0001). Subsequently, we used immunohistochemistry to examine TRPV4 expression in COH rats. As shown in Fig. 2C, weak fluorescent signals indicative of TRPV4 expression were detected in retinal sections from unoperated eyes (control) (Fig. 2Ca1). Fluorescent signals indicative of TRPV4 expression were increased at G1w, G2w, and G3w (Fig. 2Cb1, c1, d1). Quantification of these fluorescent signals demonstrated that elevated IOP increased the expression of TRPV4 in retinas (Fig. 2E, *n* = 4, respectively, *p* < 0.001 vs control). Our previous study showed that TRPV4 was widely distributed throughout the rat retina, particularly in the GCL and throughout the plexiform layer (PL) [33]. In the present study, double labeling with glutamine synthetase (GS), a Müller cell marker, revealed that TRPV4 was colocalized with Müller cells and that elevated IOP could cause enhanced expression of TRPV4 in Müller cells (Fig. 2Cb3, c3, d3).

### TRPV4 activation promotes RGC apoptosis and reduces RGC function, whereas TRPV4 inhibition is protective

**Fig. 3:**
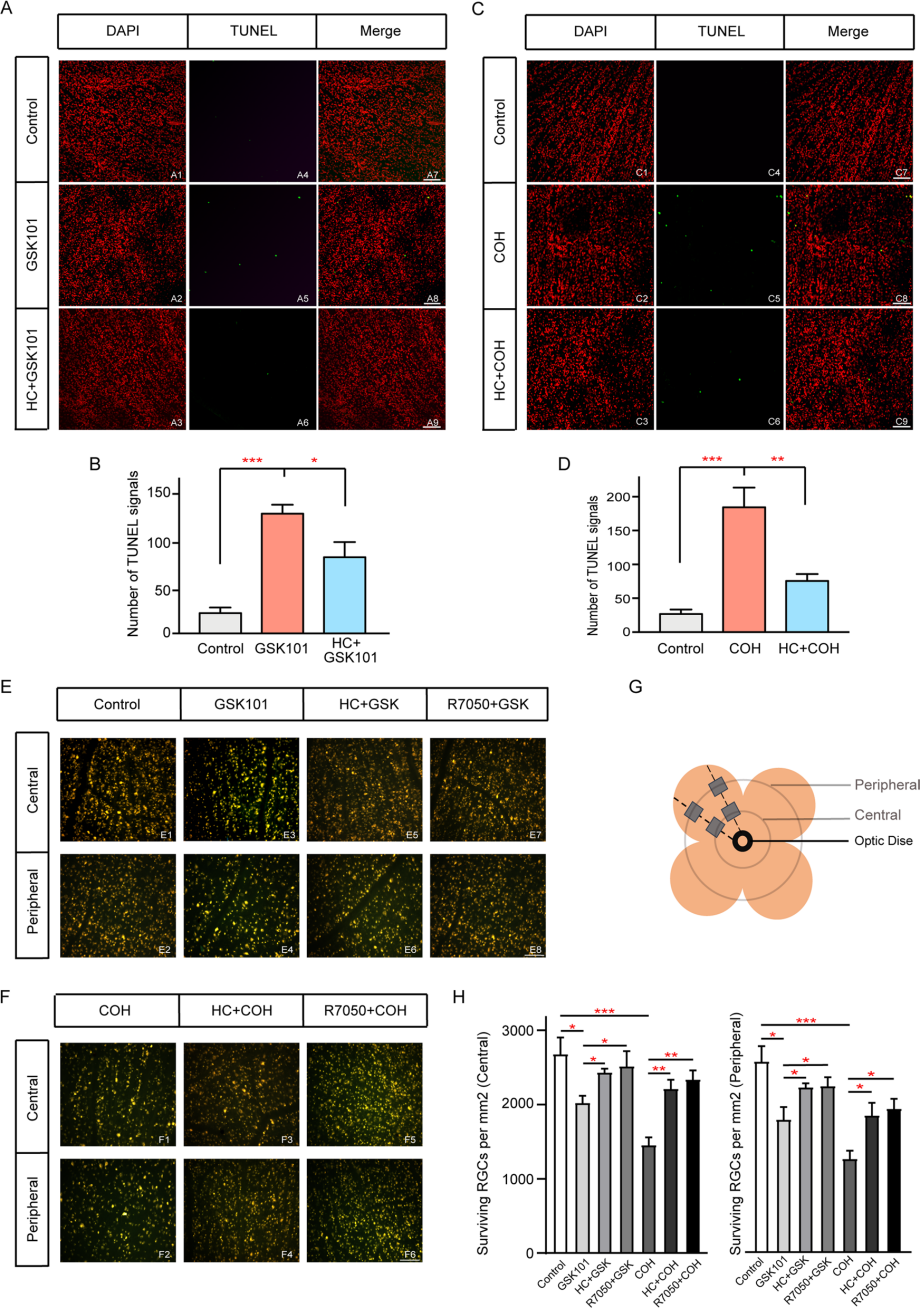
Effects of GSK101 on retinal ganglion cell (RGC) apoptosis and survival. **A** TRPV4 activation leads to enhanced RGC apoptosis. **A1**–**A3** 4′,6-diamidino-2-phenylindole (DAPI) staining in saline-injected (control) (**A1**), GSK101-injected (GSK101) (**A2**), and HC-067 with GSK101 (HC) (**A3**) retinas at 1 week after injection in the regions at angle 0°. Images were acquired from whole flat-mounted retinal preparations. **A4**–**A6**, Counterstained images with TUNEL staining detection of RGC apoptosis (green). **A7**–**A9**, Merged images of corresponding TUNEL and DAPI images. Scale bar: 50 µm. **B** Bar chart summarizing mean numbers of TUNEL-positive cells in each retina under different conditions. *n* = 5 for all groups. **C** Pre-application of HC-067 reduces RGC apoptosis in COH retinas. **C1**–**C3** DAPI staining in sham-operated (control) (**C1**), COH (**C2**), and HC-067 with COH (HC + COH) (**C3**) retinas at G2w. **C4**–**C6** Counterstained images with TUNEL staining detection of RGC apoptosis (green). **C7**–**C9** Merged images of corresponding TUNEL and DAPI images. Scale bar, 50 μm. **D** Bar chart summarizing mean numbers of TUNEL-positive cells in each retina under different conditions. *n* = 6 for all groups. *p < 0.05, **p < 0.01, and ***p < 0.001. **E**, **F** FluoroGold labeling images of surviving RGCs in flat-mounted retinas as shown in the central and peripheral panels, photographed at low magnification (20 ×). Fluorescence micrographs of flat-mounted retinas depicting FluoroGold-labeled RGCs in control (**E1**, **E2**), 1 week after GSK101 injection (**E3**, **E4**), 1 week after GSK101 plus HC-067 injection (**E5,** **E6**), 1 week after GSK101 plus R7050 injection (**E7**, **E8**), glaucoma 2 weeks (COH, **F1**, **F2**), HC-067 plus COH (**F3**, **F4**), and R7050 plus COH (**F5**, **F6**) retinas. Scale bar: 50 µm. **G** Schematic diagram showing different retinal regions. **H** Quantitative analysis of the density (cells/mm^2^) of surviving RGCs under different conditions in the central and peripheral regions

RGC apoptosis is a major cause of glaucoma-related irreversible blindness caused by glaucoma. Here, we used TUNEL assays to investigate whether changes in TRPV4 expression contributed to RGC apoptosis in COH retinas. Figure 3A, B shows representative images of cell apoptosis in a flat-mounted retina at a consistent angle (0°) under different experimental conditions. The mean number of TUNEL-positive signals in each retina was increased at 1 week after injection of GSK101, a TRPV4 agonist (10 µM, Fig. 3A5); this sharply contrasted with the low level of TUNEL-positive signals present in saline-injected (control) retinas (Fig. 3A4). The mean number of TUNEL-positive cells per retina was significantly higher in GSK101-injected retinas than in saline-injected retinas (121.8 ± 9.2 vs 21.4 ± 4.7, *n* = 5, *p* < 0.0001). Furthermore, pre-injections of a TRPV4 antagonist, HC-067 (10 µM), were sufficient to diminish the GSK101-induced effect; the mean number of TUNEL-positive signals in each retina was reduced to 77.4 ± 15.5 (*n* = 5, *p* = 0.0127 vs GSK101-injected retinas, Fig. 3A6, B).

Considering that RGC apoptosis was first detected after 2 weeks of glaucoma modeling [36], we performed TUNEL assays at G2w. As shown in Fig. 3C, the mean number of TUNEL-positive signals in each retina was increased at G2w after IOP elevation (Fig. 3C5); this sharply contrasted with the low level of TUNEL-positive signals present in sham-operated (control) retinas (Fig. 3C4). The mean number of TUNEL-positive cells in each retina at G2w was significantly higher than the mean number in sham-operated retinas (184.5 ± 30.0 vs 26.7 ± 6.7, *n* = 6, *p* < 0.0001). Pre-administration of HC-067 could significantly reduce the TUNEL-positive signals in COH model; the mean number of TUNEL-positive signals in each retina was reduced to 75.5 ± 10.2 (*n* = 6, *p* = 0.0014 vs COH alone, Fig. 3C6, D). These results suggested that TRPV4 inhibition led to neuroprotection after COH.

Because TUNEL-positive cells in flat-mounted retinas may include some astrocytes and displaced amacrine cells (in addition to RGCs), we counted the numbers of FluoroGold-labeled surviving RGCs to evaluate the effects of TRPV4 activation in RGCs. As shown in Fig. 3E–G, the number of labeled RGCs was significantly decreased in GSK101-pre-injected retinas, while pre-treatment with HC-067 abolished the damaging effects of GSK101 in both central and peripheral regions (in the central region, GSK101: 2024 ± 118.4/mm^2^ vs control: 2687 ± 240.2/mm^2^, *p* = 0.033; HC + GSK101: 2433 ± 49.6/mm^2^ vs GSK101, *p* = 0.028; in the peripheral region, GSK101: 1794 ± 183.7/mm^2^ vs control: 2576 ± 222.2/mm^2^, *p* = 0.037; HC + GSK101 2315 ± 56.81/mm^2^ vs GSK101, *p* = 0.035; *n* = 4–6, Fig. 3E1–E6). Moreover, the number of surviving RGCs increased in glaucomatous retinas with HC-067 pre-injection at G2w, compared with glaucoma alone (in the central region, COH: 1462 ± 120.6/mm^2^ vs control, *p* < 0.001; HC + COH 2221 ± 136.0/mm^2^ vs COH, *p* = 0.003; in the peripheral region, COH: 1278 ± 109.8/mm^2^ vs control, *p* < 0.001; HC + COH: 1815 ± 135.9/mm^2^ vs COH, *p* = 0.015, *n* = 4–6, Fig. 3F1–F12). Taken together, these results indicated that TRPV4 activation was involved in elevated IOP-induced RGC apoptosis.

**Fig. 4:**
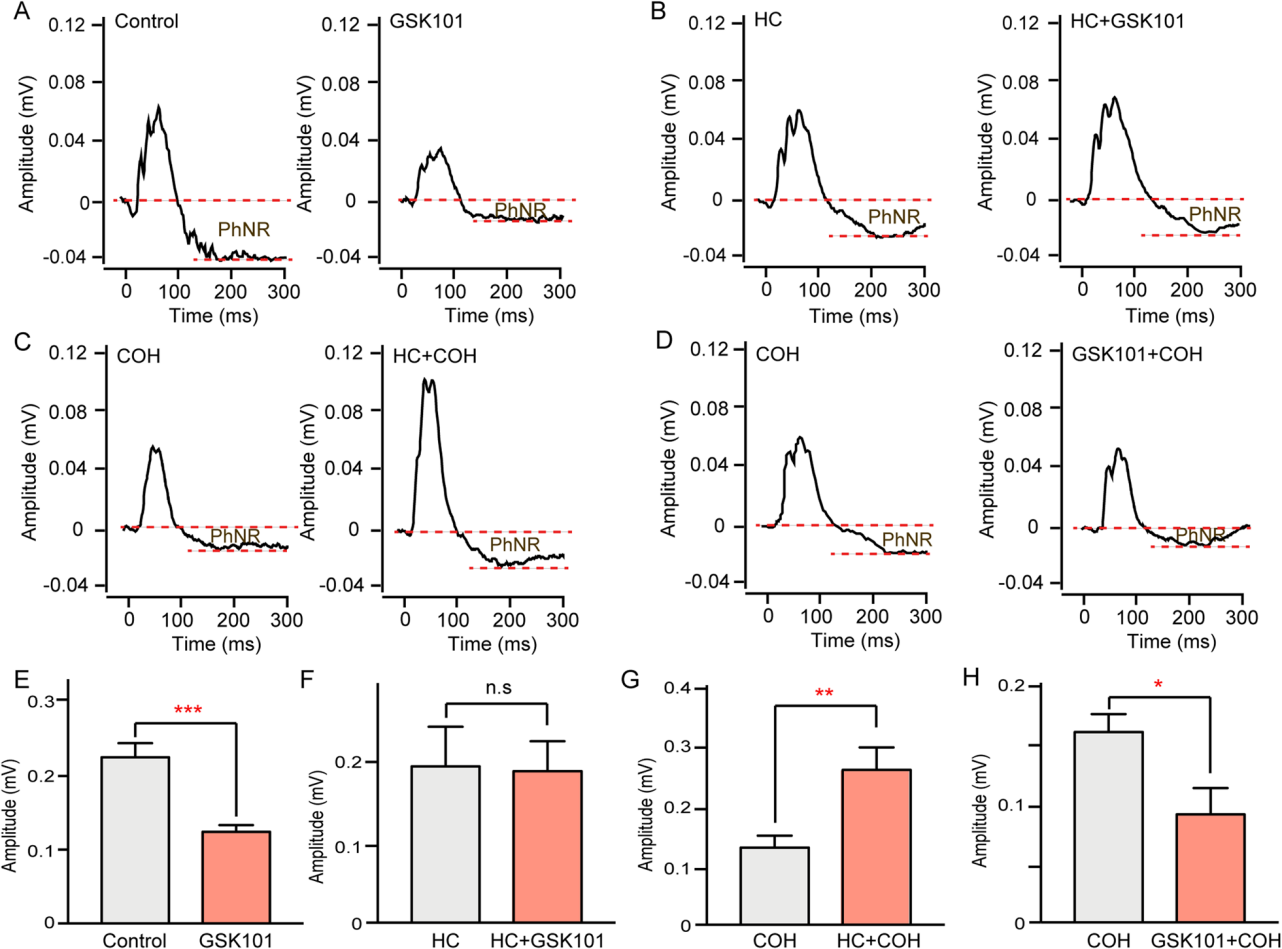
Effects of GSK101 on retinal ganglion cell (RGC) photopic negative response (PhNR). **A** Representative PhNR of 1 week retina after saline injection (control) and GSK101 injection (GSK101). **B** Representative PhNR of 1 week retina after HC-067 plus saline injection (HC) and HC-067 plus GSK101 injection (HC + GSK101). **C** Representative PhNR of COH and HC-067 with COH (HC + COH) retinas at G2w. **D** Representative PhNR of COH and GSK101 with COH (GSK101 + COH) retinas at G2w. **E**–**H** Data analyses of PhNR amplitudes under different conditions, *n* = 4–7, *p < 0.05, **p < 0.01, and ***p < 0.001

Next, we examined the effects of TRPV4 activation on RGC function. The PhNR amplitude was significantly lower in the GSK101-injected group than in the saline-injected group (59.1% ± 8.2% control, *n* = 7, *p* = 0.017, Fig. 4A, E), while the PhNR amplitude did not significantly differ between the HC-067 plus GSK101-injected group and the HC-067 plus saline-injected group (*n* = 4, *p* = 0.925, Fig. 4B, F). HC-067 administration significantly increased the PhNR amplitude in the COH group (193.5% ± 25.35% of COH, *n* = 5, *p* = 0.009, Fig. 4C, G); GSK101 administration led to further reduction of PhNR amplitude (56.7% ± 14.12% of COH, *n* = 5, *p* = 0.0342, Fig. 4D, H). These results indicated that TRPV4 activation could reduce RGC function. In scotopic ERG, the a-wave reflects retinal photoreceptor activity and the b-wave reflects inner nuclear layer (INL) activity (e.g., bipolar cells and Müller cells) [39–42]. Our scotopic ERG findings showed that the a-wave and b-wave amplitudes were significantly reduced to 62.1% and 52.2% of control amplitudes (*n* = 4, *p* < 0.05 vs control, Additional file 1: Fig. S1A, B). These results indicated that—in addition to RGC function—TRPV4 activation may disrupt retinal photoreceptor function and INL function.

### Functional TRPV4 is expressed in Müller cells

**Fig. 5:**
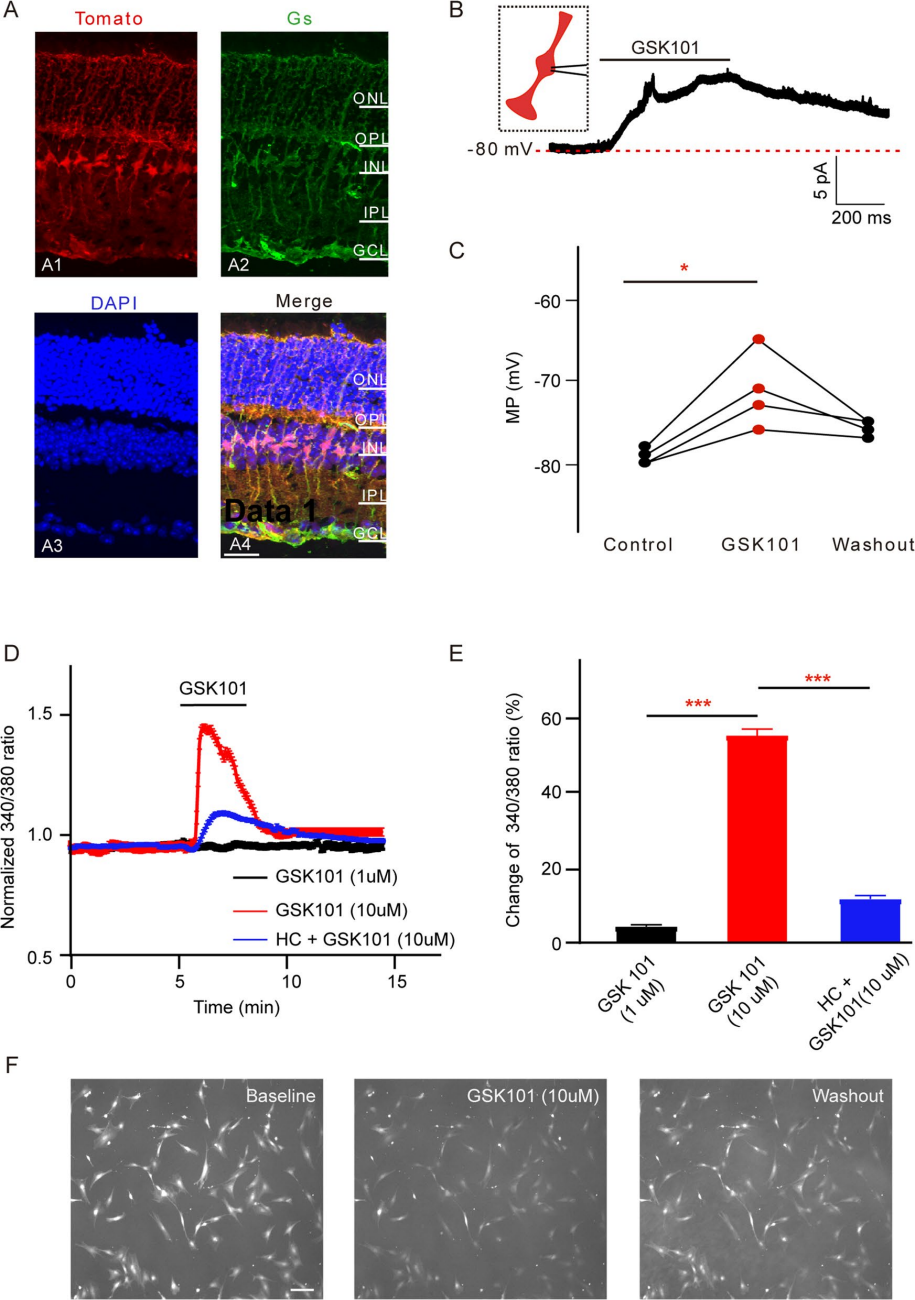
Müller cells express functional TRPV4. **A** Immunofluorescence images show that cells with red autofluorescence are Müller cells. **A1** Immunofluorescence images show cells with red autofluorescence in rat retinal vertical slices acquired from TdTomato transgenic mouse retina (Tomato). **A2** Immunofluorescence images show glutamine synthetase (GS) protein staining (green) in the slices depicted in **A1**. **A3** Immunofluorescence images show DAPI (blue) staining in the slices depicted in **A1**. **A4** shows merged images. Scale bar: 50 µm. **B** Representative trace recorded from a Müller cell identified by spontaneous red fluorescence, showing that perfusion with GSK101 (10 μM) caused significant depolarization of Müller membrane potential. **C** Bar chart showing GSK101-induced depolarization of Müller cell membrane potential. *n* = 4. *p < 0.05 vs control. **D** Representative trace from cultured Müller cells loaded with Fura-2 and exposed to GSK101 (1 µM, black trace; 10 µM, red trace) or GSK101 in conjunction with HC-06 (blue trace). **E** Change of the 340/380 ratio in cultured Müller cells treated with either GSK101 alone or GSK101 in the presence of HC-06 (*n* = 69–125). ***p < 0.001. **F** Representative cultured Müller cells showing the fluorescence changes in the presence of GSK101 (10 µM). Scale bar: 50 µm

Expression of functional TRPV4 was assessed using electrophysiology and calcium imaging. TdTomato transgenic mouse retinal Müller cells showed spontaneous red fluorescence, which colocalized with GS green fluorescence (Fig. 5A4). The application of GSK101 (10 µM) caused Müller cell membrane depolarization by 10.1% (control: − 79.3 ± 0.48 mV vs GSK101: − 71.25 ± 2.32 mV, *n* = 4, *p* = 0.034, Fig. 5B, C).

Considering that TRPV4 is a nonselective cation channel with a slight preference for calcium, we evaluated the changes in calcium homeostasis associated with TRPV4 activation in Müller cells. Cells were loaded with the Ca^2+^ indicator Fura-2AM, then stimulated with GSK101 (1 µM and 10 µM). As shown in Fig. 5D, at 1 µM, GSK101 treatment did not affect the 340/380 nm ratio; at 10 µM, GSK101 treatment could increase the 340/380 nm ratio by 55.3% (*n* = 69). These GSK101-evoked signals were decreased by treatment with the selective antagonist HC-06 (10 µM; HC + GSK101 treatment increased the 340/380 nm ratio by 11.7%, *n* = 125, *p* < 0.001 vs. GSK101 10 µM increasement, Fig. 5D–F). Overall, the results implied that functional TRPV4 is expressed in Müller cells.

### TRPV4 activation induces Müller cell gliosis

**Fig. 6:**
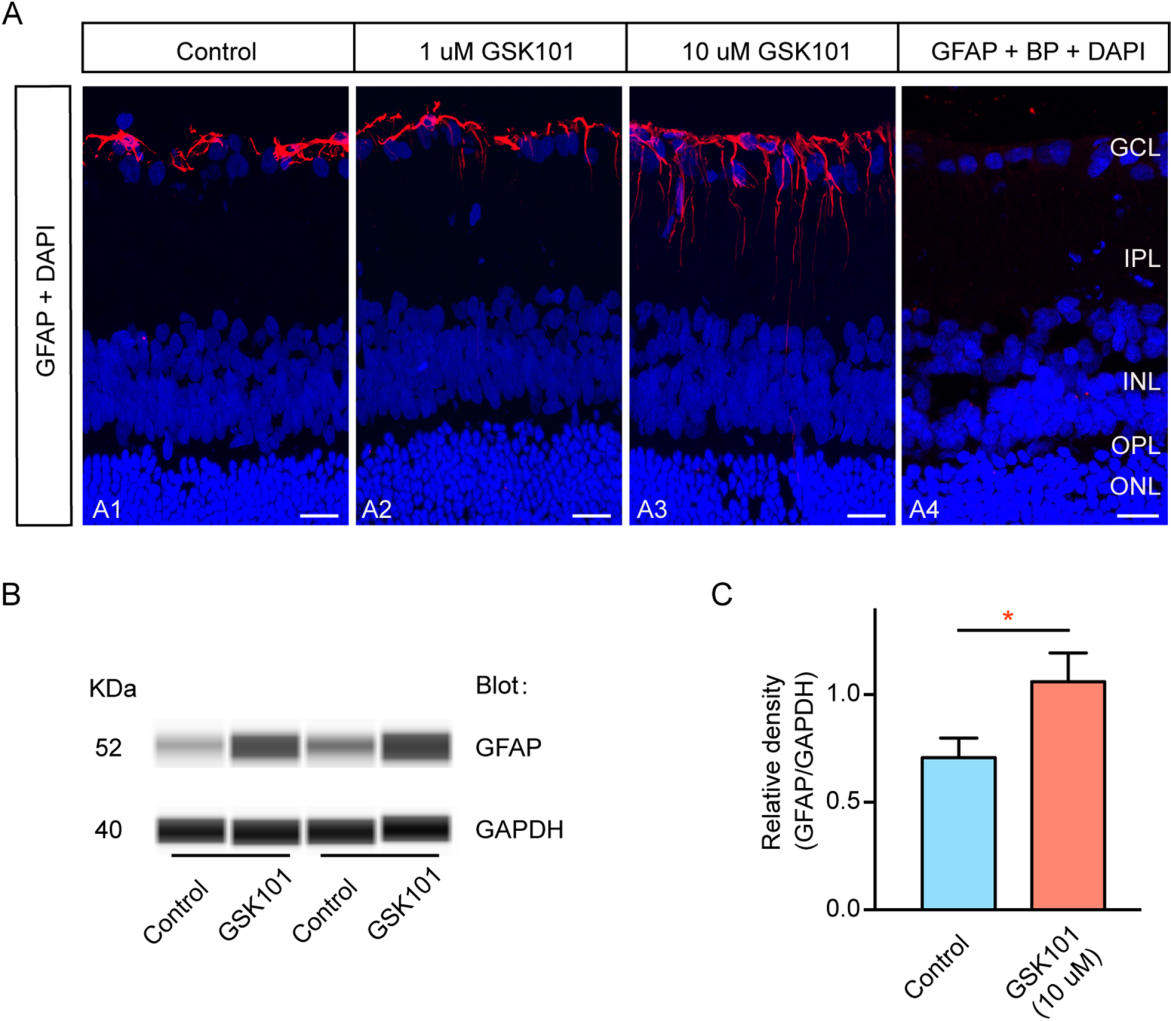
TRPV4 activation enhances the expression of GFAP. **A** Immunofluorescence images show GFAP protein expression profiles in rat retinal vertical slices acquired from sham-operated retinas (saline-injected; control) (**A1**), 1 µM GSK101-injected retinas (**A2**), and 10 µM GSK101-injected retinas (**A3**). Retinas that received no GFAP antibody served as negative controls (**A4**). Double immunofluorescence staining showing GFAP expression when the GFAP antibody was pre-adsorbed with its blocking peptide (BP) (**A5**). Scale bar: 20 µm. **B** Representative immunoblots showing changes in GFAP protein levels in control and 10 µM GSK101-injected retinas. **C** Bar chart summarizing mean expression levels of GFAP under different conditions. *n* = 6. *p < 0.05 vs control

Next, we studied the mechanism underlying TRPV4 activation-induced RGC apoptosis. Considering the expression of functional TRPV4 and the increased expression of TRPV4 by Müller cells in our COH model (Figs. 2Ca3–d3, 5), we mainly focused on TRPV4 function in Müller cells. As shown in Fig. 6A, in normal saline-injected (control) sections, GFAP expression was detected close to the GCL (Fig. 6A1). Upon intravitreal injection of 1 µM GSK101, the GFAP expression slightly increased (Fig. 6A2), while 10 µM GSK101 led to significantly increased GFAP expression (Fig. 6A3). Immunoblotting confirmed that the injection of 10 µM GSK101 significantly enhanced the expression of GFAP in retinas (149.72% ± 18.93%, *n* = 6, *p* = 0.017, Fig. 6B, C). Therefore, 10 µM GSK101 was used in subsequent experiments. Moreover, these results implied that TRPV4 activation could induce Müller cell gliosis.

### TRPV4 activation induces TNF-a production

**Fig. 7:**
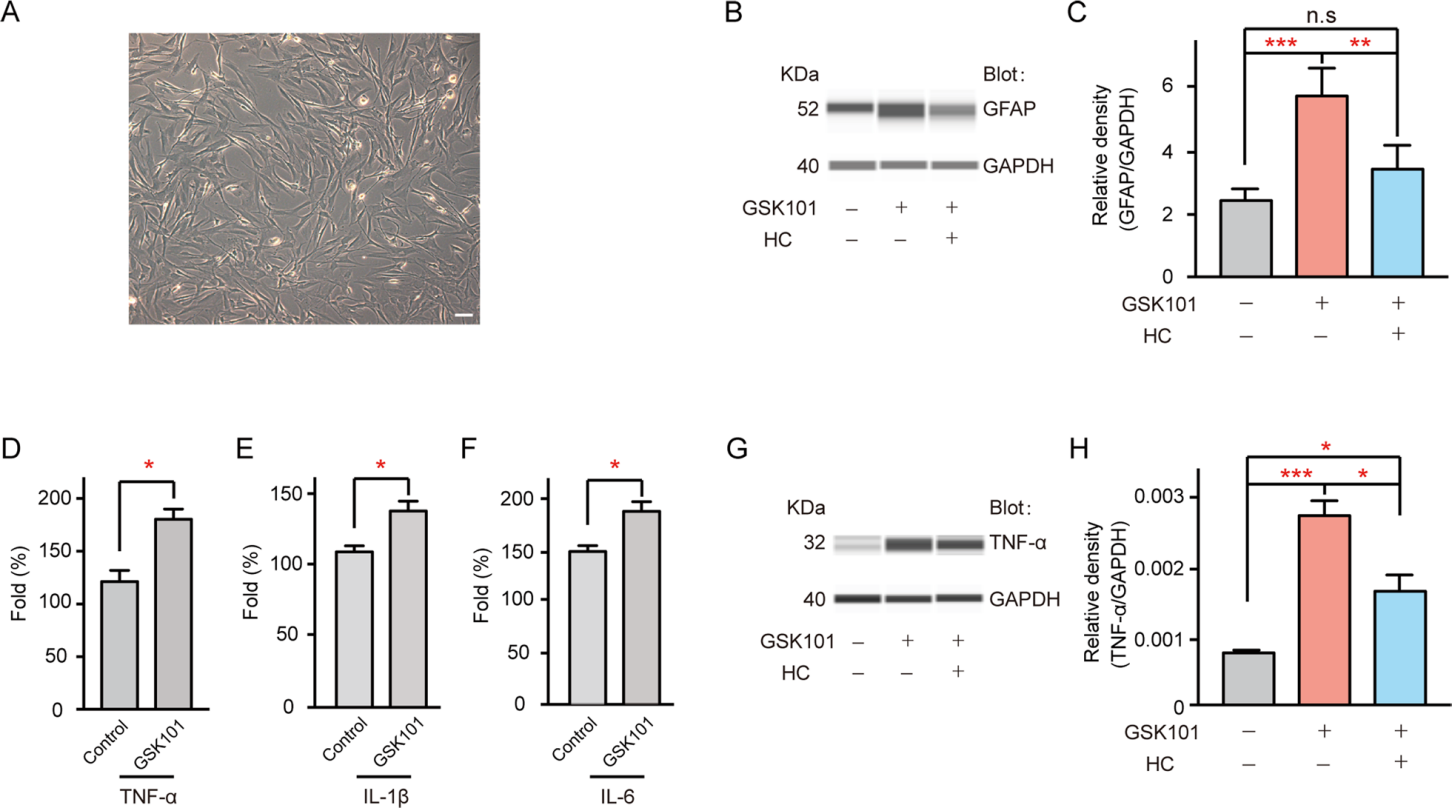
TRPV4 activation enhances TNF-α production in cultured Müller cells. **A** Morphology of cultured Müller cells. Scale bar: 50 µm. **B** GSK101 treatment enhanced GFAP protein levels in cultured Müller cells. *n* = 4. **p < 0.01 and ***p < 0.001. **C** Cumulative changes in GFAP protein levels in control and GSK101 treatment groups. **D**–**F** Cumulative changes in mRNA levels of TNF-α (**D**), IL-1β (**E**), and IL-6 (**F**) in Müller cell extracts obtained after saline treatment (control) or GSK101 treatment for 24 h. Relative abundances of mRNA were quantified using the 2^−ΔΔct^ calculation method and are expressed as fold changes. *n* = 3 for all groups. *p < 0.05 vs control. **G**, GSK101 treatment led to enhancement of TNF-α protein level. **H** Cumulative changes in TNF-α protein levels in control and GSK101 treatment groups. *n* = 6. *p < 0.05 and ***p < 0.001

Here, we examined whether TRPV4 activation induces cell gliosis in cultured Müller cells (Fig. 7A). As shown in Fig. 7B, the application of GSK101 enhanced GFAP expression (232.4% ± 30.9% of control, *n* = 4, *p* < 0.001), while pre-application of HC-067 tended to reduce this effect (141.53% ± 28.07% of control, *n* = 4, *p* = 0.176, Fig. 7C). These results are consistent with the findings in Fig. 6 and suggest that TRPV4 activation could induce Müller cell gliosis.

In glaucomatous retinas, activated Müller cells may release cytotoxic substances. Thus, we used the real-time polymerase chain reaction technique to examine changes in the mRNA levels of tumor necrosis factor α (TNF-α), interleukin-1β (IL-1β, a proinflammatory cytokine) and interleukin-6 (IL-6, a proinflammatory cytokine) in cultured Müller cells after GSK101 treatment. As shown in Fig. 7D–F, the mRNA levels of TNF-α, IL-1β, and IL-6 were higher in GSK101-treated Müller cells than in control cells (TNF-α: 132.8% ± 10.6% of control, *n* = 3, *p* = 0.0147; IL-1β: 126.2% ± 6.3% of control, *n* = 3, *p* = 0.0236; IL-6: 149.5% ± 13.9% of control, *n* = 3, *p* = 0.0442), indicating enhanced production of proinflammatory cytokines. Similarly, the protein level of TNF-α was increased in the GSK101 treatment group (377.39% ± 41.08% of control, *n* = 6, *p* < 0.001); this effect was slightly attenuated by treatment with HC-067 (229.59% ± 30.34% of control, *n* = 6, *p* = 0.019 vs GSK101, *p* = 0.041 vs control, Fig. 7G, H).

**Fig. 8:**
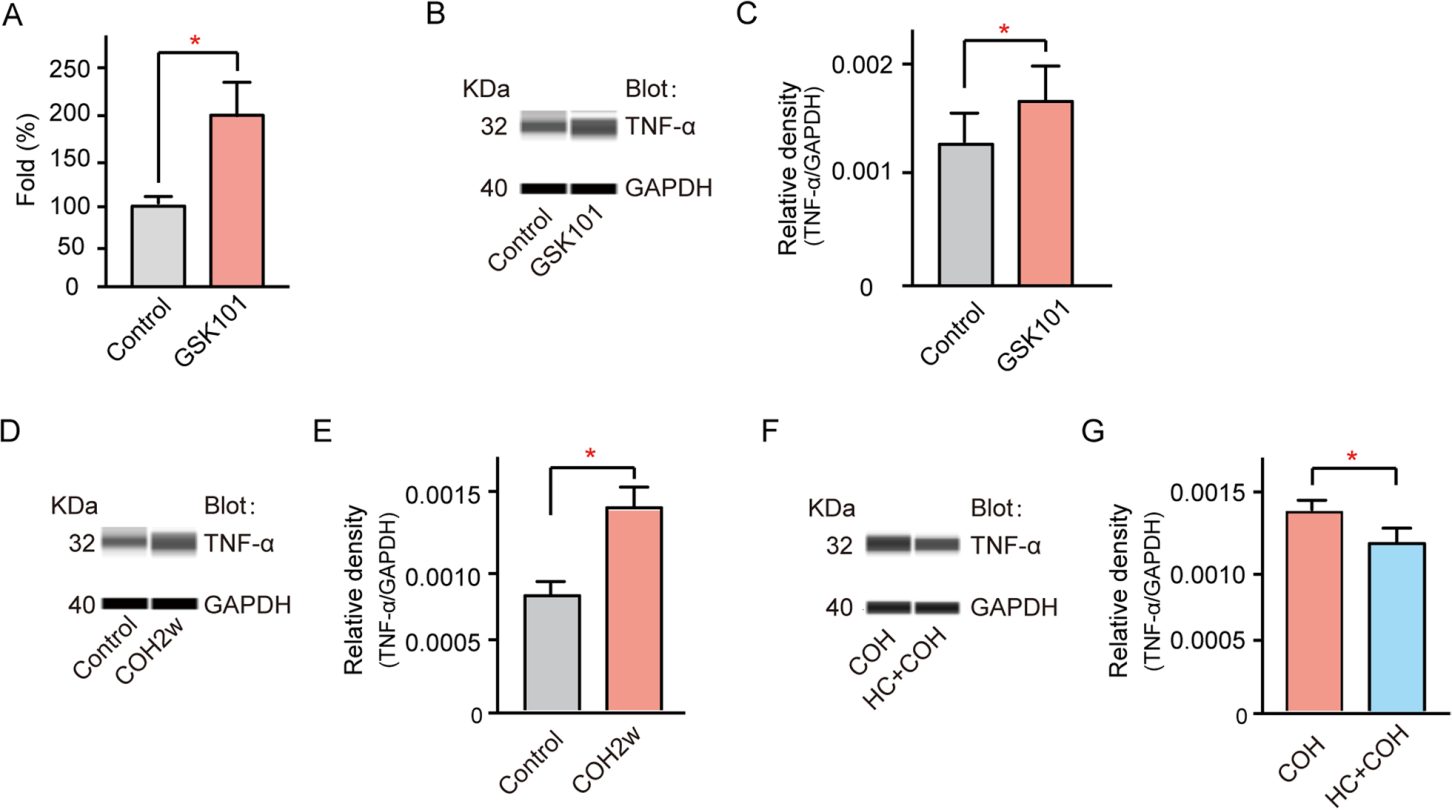
TRPV4 activation enhances TNF-α production in retinal tissues. **A** Cumulative changes in TNF-α mRNA levels in saline-injected retinas (control) and retinas with GSK101 injection at 1 week. *n* = 5, *p < 0.05 vs control. **B** GSK101 treatment enhanced TNF-α protein levels in retinas with GSK101 injection at 1 week. **C** Bar chart summarizing mean expression levels of TNF-α under different conditions. *n* = 3. *p < 0.05. **D** Representative immunoblots showing changes in TNF-α protein levels in control and COH retinal extracts at G2w. **E** Bar chart summarizing mean expression levels of TNF-α under different conditions. *n* = 3. *p < 0.05. **F** Representative immunoblots showing changes in TNF-α protein levels in COH, and HC-067 plus COH retinas at 2 weeks after establishment of the COH model. **G** Bar chart summarizing mean expression levels of TNF-α under different conditions. *n* = 8. *p < 0.05

TNF-α expression was examined in normal retinas after intravitreal injection of GSK101. In these experiments, the TNF-α mRNA level was significantly increased to 206.36% ± 35.6% of control (*n* = 5, *p* = 0.0299, Fig. 8A). Similarly, the TNF-α protein level was increased to 133.25% ± 22.78% of control (*n* = 3, *p* = 0.0426, Fig. 8B, C). Notably, elevated IOP led to an increased TNF-α protein level (142.4% ± 13.7% of control, *n* = 3, *p* = 0.0148, Fig. 8D, E), and pre-administration of HC-067 could significantly reduce the TNF-α protein level in our COH model (76.14% ± 13.7% of COH, *n* = 4, *p* = 0.0454, Fig. 8F, G). These results suggested that TRPV4 activation induces TNF-α production.

### TRPV4 agonist application influences JAK2–STAT3 signaling and NF-kB p65 activity in Müller cells

**Fig. 9:**
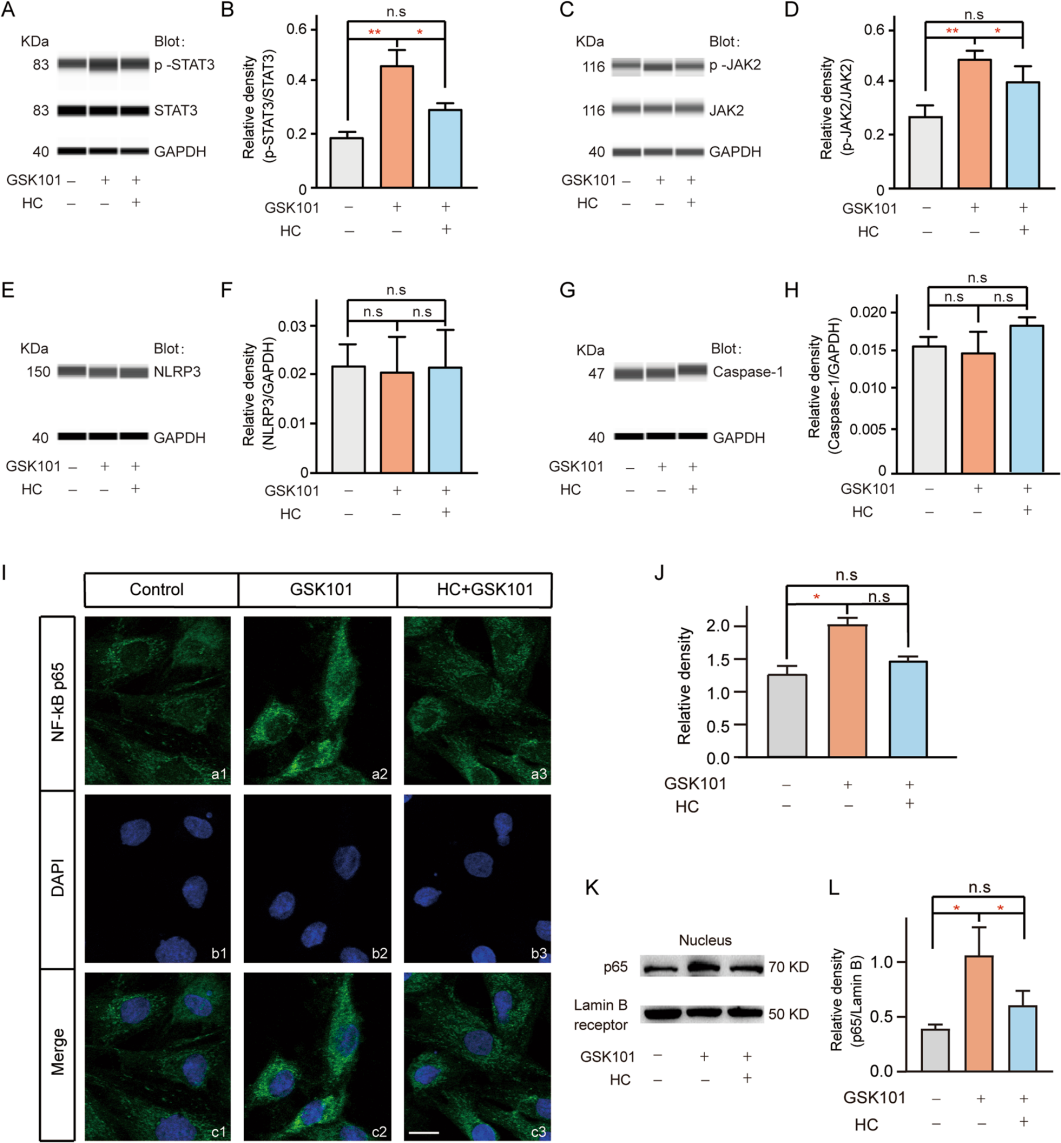
TRPV4 activation led to increased phosphorylation of JAK2 and STAT3, thereby inducing NF-κB p65 translocation from the cytoplasm into the nucleus. **A**, **C** Immunoblotting analysis showing that GSK101 treatment increased the phosphorylation of STAT3 and JAK2, compared with the control group, but had no effect on the protein levels of JAK2 and STAT3 (**C**). **B**, **D** Bar charts summarizing mean expression levels of phosphorylated STAT3/STAT (**B**) and phosphorylated JAK2/JAK2 (**D**) under different conditions. *n* = 3 for all groups, *p < 0.05 and **p < 0.01. **E**, **G** Immunoblotting analysis showing that GSK101 treatment had no effect on the protein levels of NLRP3 (**E**) and caspase-1 (**G**), compared with the control group. **F**, **H** Bar charts summarizing mean expression levels of NLRP3 (**F**) and caspase-1 (**H**) under different conditions. *n* = 3 for all groups. **I** Confocal laser microphotographs of cultured Müller cells, stained with an antibody against NF-κB p65 (green), showing changes in NF-κB p65 protein expression in saline treatment (control), GSK101 treatment (GSK101), and HC Plus GSK101 (HC + GSK101) groups (**a1**–**a3**). **b1**–**b3** are DAPI images. **c1**–**c3** are merged images. Scale bar, 10 μm (for all images). **J** Bar graph summarizing mean density of NF-κB p65 immunofluorescence in Müller cells under different conditions. *n* = 3 for all groups. *p < 0.05. **K** Immunoblotting analysis showing that GSK101 treatment enhanced the expression of NF-κB p65 in Müller cell nuclei, compared with the control and preapplication of HC groups. **L** Bar chart summarizing mean expression levels of NF-κB p65 under different conditions. *n* = 5, *p < 0.05 and **p < 0.01

Immunoblotting was used to confirm whether TRPV4 induced JAK2–STAT3 signaling after application of GSK101. Immunoblotting showed that the phosphorylation levels of STAT3 and JAK2 were significantly elevated after GSK101 application, while the levels of STAT3 and JAK2 did not change (phosphorylation of STAT3/STAT3: 224.9% ± 25.0% of control, *n* = 3, *p* = 0.0029; phosphorylation of JAK2/JAK: 148.0% ± 8.52% of control, *n* = 3, *p* = 0.004); these effects could be reversed by pre-application of the TRPV4 antagonist HC-067 (Fig. 9A–D).

In addition, we examined changes in the expression levels of NLRP3 inflammasome components (NLRP3 and caspase-1) in cultured Müller cells that had been subjected to GSK101 treatment. As shown in Fig. 9E–H, the expression levels of NLRP3 and caspase-1 did not change at 24 h after GSK101 treatment (NLRP3: *n* = 3, *p* = 0.9907, Fig. 9E, F; caspase-1: *n* = 3, *p* = 0.0956, Fig. 9G, H). These results indicate that NLRP3 inflammasome activation may not be involved in the TRPV4 activation-induced enhancement of TNF-α expression.

We then explored whether the transcription factor nuclear factor-kappa B (NF-κB) influences Müller cell-mediated changes in inflammatory cytokines. In normal cultured Müller cells, faint fluorescence indicative of NF-κB p65 expression was evident in the cytosol; the total fluorescence was significantly enhanced in the GSK101 treatment group (169.2% ± 8.1% of control, *n* = 3, *p* = 0.016 vs control, Fig. 9I, J). Furthermore, fluorescence indicative of NF-κB p65 expression was detected in the nucleus in the GSK101 treatment group, suggesting translocation from the cytosol to the nucleus (Fig. 9I). Changes in NF-κB p65 expression in the nucleus were confirmed by immunoblotting (GSK101: 297.8% ± 89.3% of control, *n* = 4, *p* = 0.004 vs control, Fig. 9K, L).

### TRPV4 activation could lead to elevated TNFR1 expression in RGCs

**Fig. 10:**
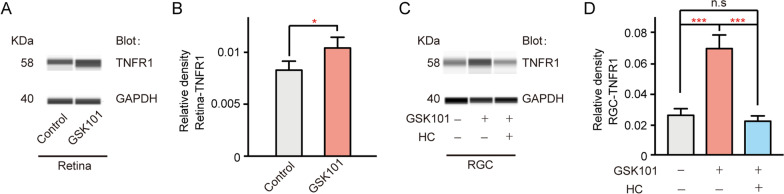
TRPV4 activation led to enhanced expression of TNF receptor 1 (TNFR1). **A** Immunoblotting analysis showing that pre-injection of GSK101 enhanced the expression of TNFR1 in the retina, compared with the control group. **B** Bar chart summarizing mean expression levels of TNFR1 under different conditions. *n* = 5, *p* < 0.01. **C** Immunoblotting analysis showing that GSK101 treatment enhanced the expression of TNFR1 in cultured RGCs, compared with the control group. **D** Bar chart summarizing mean expression levels of TNFR1 under different conditions. *n* = 3, ***p < 0.001

TRPV4 is also expressed in RGCs [33]. Considering the damage effects of TNF receptor 1 (TNFR1) on RGCs in many eye diseases [43], we explored the effects of TRPV4 activation on TNFR1 expression. Notably, pre-injection of GSK101 could increase the expression of TNFR1 throughout the retina (125.89% ± 5.09% of control, *n* = 5, *p* = 0.033, Fig. 10A, B). In cultured RGCs, we found that GSK101 application could increase the expression of TNFR1 to 264.25% ± 27.48% of control (*n* = 3, *p* < 0.001 vs control); this effect was attenuated by HC-067-induced TRPV4 inhibition (*p* = 0.524 vs control, Fig. 10C, D). These results suggest that the TRPV4 activation-induced enhancements of TNF-a release and TNFR1 expression in RGCs could be involved in RGC apoptosis in glaucoma.

### TNF-α inhibition reduces TRPV4-mediated retinal cell apoptosis

**Fig. 11:**
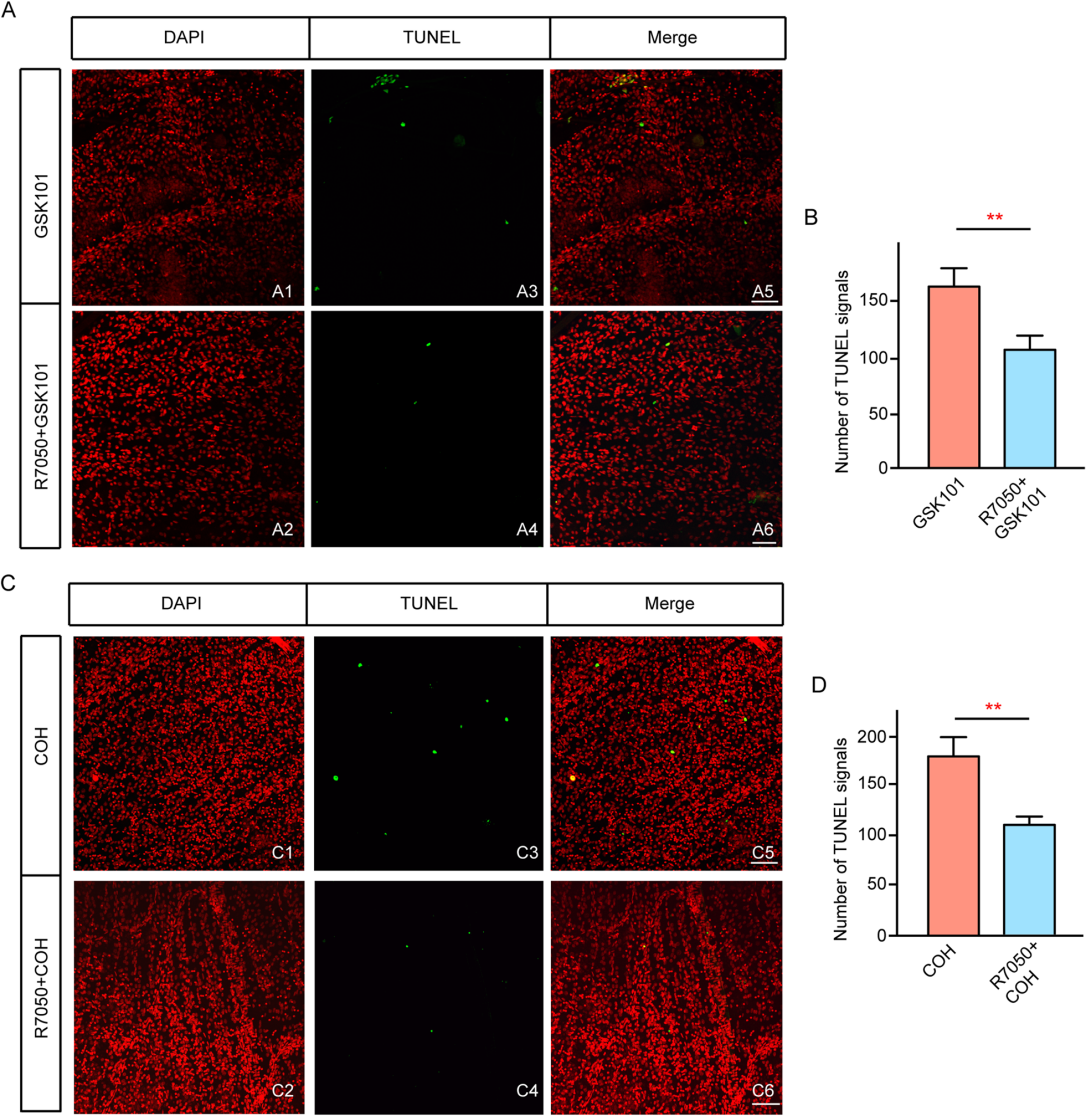
TNF-α inhibition reduces TRPV4-mediated retinal cell apoptosis. **A1**–**A2** DAPI staining in GSK101-injected (**A1**) and R7050 with GSK101-injected (R7050 + GSK101) (**A2**) whole flat-mounted retinas at 7 days after injections in the regions at angle 0°. **A3**–**A4** Counterstained images with TUNEL staining detection of cell apoptosis (green). **A5**–**A6** Merged images of corresponding TUNEL and DAPI images. Scale bar, 50 µm (for all images). **B** Bar chart summarizing mean numbers of TUNEL-positive cells in each retina under different conditions. R7050 (1 µM, 2 μl) was pre-injected 1 day before the GSK101 injection. *n* = 5. **p < 0.01 vs control (for all images). **C1**–**C2** DAPI staining in COH (**C1**) and R7050 with COH (R7050 + COH) (**C2**) whole flat-mounted retinas at G2w in the regions at angle 0°. **C3**–**C4**, Counterstained images with TUNEL staining detection of cell apoptosis (green). **C5**–**C6**, Merged images of corresponding TUNEL and DAPI images. Scale bar, 50 µm (for all images). **D** Bar chart summarizing mean numbers of TUNEL-positive cells in each retina under different conditions. R7050 (1 µM, 2 μl) was pre-injected 1 day before COH modeling. *n* = 5. **p < 0.01 vs control

Finally, we performed intravitreal injection of an inhibitor of soluble TNF-α, R7050 (1 µM in 2 μl), prior to GSK101 injection. Figure 11 shows that intravitreal injection of R7050 significantly reduced the number of TUNEL-positive signals in GSK101-injected retinas (Fig. 11A3, A4). The mean number of TUNEL-positive cells per retina was significantly higher in GSK101-injected retinas than in retinas with pre-administration of R7050 (162.2 ± 17.4 vs 106.6 ± 13.8, *n* = 5, *p* = 0.007), indicating that R7050 reduced the GSK101-mediated effect. In another experiment, R7050 was intravitreally injected 1 day before establishment of the COH model; these pre-injections of R7050 caused a significant reduction in the number of TUNEL-positive cells (Fig. 11C); the mean number of TUNEL-positive signals in each retina was reduced to 108.8 ± 12.7 (*n* = 5), which was considerably lower than the mean number in COH retinas at G2w (186.0 ± 16.2, *n* = 5, *p* = 0.004). We also counted the number of FluoroGold-labeled surviving RGCs to evaluate the effects of TNF-α on RGCs. As shown in Fig. 3E7, E8, there were significantly more labeled RGCs in R7050-pre-injected retinas, compared with retinas that underwent GSK101 injection alone (in the central region, R7050 + GSK101: 2596 ± 160.0/mm^2^, *p* = 0.019 vs GSK101 injection alone; in the peripheral region, R7050 + GSK101: 1815 ± 135.9/mm^2^, *p* = 0.002 vs GSK101 injection alone; Fig. 3E7, E8). Moreover, the number of surviving RGCs increased in glaucomatous retinas (G2w) with R7050 pre-injection, compared with glaucoma alone (in the central region, R7050 + COH: 1462 ± 120.6/mm^2^, *p* = 0.043 vs COH alone; in the peripheral region, R7050 + COH: 1949 ± 203.9/mm^2^, *p* = 0.013 vs COH alone, Fig. 3F5 and F6). These results suggest that the pre-inhibition of TNF-α by injection of R7050 could reduce elevated IOP-induced retinal cell apoptosis and increase RGC survival.

## Discussion

### Elevated IOP enhances the expression of TRPV4 protein in COH retinas

TRPV4 is widely distributed in the retina, where it is involved in various physiological and pathological processes. In RGCs, TRPV4 activation has been shown to regulate sodium currents, presynaptic inhibitory transmission, and firing rate, indicating the involvement of TRPV4 in the intrinsic properties of RGCs [18, 24, 33]. TRPV4 can also induce Müller cell gliosis by sensing changes in cell volume, while mediating fluid exchange across the blood–retina and blood–brain barriers [17, 19, 44, 45]. Furthermore, TRPV4 activation can lead to apoptosis in photoreceptor cells and RGCs, indicating that TRPV4 is involved in retinal detachment and glaucoma [46].

Glaucomatous retinopathy is strongly associated with IOP elevation, but the relationship between IOP and TRPV4 has not been revealed [1]. In our rat model of COH, we found that elevated IOP may activate TRPV4, particularly in the GCL and Müller cells (Fig.  2C). We speculate that the elevated IOP-mediated TRPV4 activation may have the following characteristics. First, during IOP elevation, the optic nerve is stretched, which may directly activate mechanosensitive TRPV4 or indirectly activate TRPV4 through other mechanosensitive channels; this mechanism requires further study [21, 22, 24]. Second, elevated IOP triggers Müller cell gliosis. The changes in cell volume and osmotic pressure caused by Müller cell activation may be sufficient to stimulate the volume and osmotic pressure-sensitive TRPV4, thereby increasing TRPV4 expression [17].

In recent studies, neuronal injury has been shown to cause changes in TRPV4 expression. For example, the expression levels of TRPV4 increase in rat hippocampi during infrasound-induced neuronal impairment; furthermore, rapidly increased TRPV4 expression disrupts endothelial cell organization during the early inflammatory phase of experimental spinal cord injury, resulting in tissue damage, vascular destabilization, blood–spinal cord barrier breakdown, and scarring [47, 48]. In the present study, we showed that TRPV4 activation could induce RGC apoptosis, whereas pre-inhibition of TRPV4 could alleviate the elevated IOP-mediated or TRPV4 activation-mediated RGC apoptosis (Fig. 3). Our study findings complement the changes in TRPV4 expression in the context of ocular hypertension; they also suggest that TRPV4 can serve as a therapeutic target in glaucoma.

### TRPV4 activation could cause RGC apoptosis through the release of inflammatory cytokines

Müller cell activation can cause RGC apoptosis in glaucoma through the release of inflammatory factors [26]. Our results showed that TRPV4 activation could enhance GFAP expression and depolarize the Müller cell membrane, indicating that TRPV4 activation may promote Müller cell gliosis and affect RGC apoptosis [26]. In addition, TRPV4 activation could directly lead to the release of inflammatory factors, particularly TNF-α, which participates in RGC apoptosis in glaucoma [43, 49]. Finally, TRPV4 inhibition led to reduced Müller cell reactivity, as well as lower overall levels of GFAP expression, TNF-α release, and RGC apoptosis; these findings implied that TRPV4 activation-mediated inflammatory factors were involved in RGC apoptosis in glaucoma.

Multiple studies have shown that TRPV4 activation enhances the production and release of inflammatory cytokines in the central nervous system and retina. Notably, a TRPV4 agonist has been reported to reduce the lipopolysaccharide-induced microglial release of TNF-α [50]. In contrast, significant infrasound-induced astrocytic and microglial activation has been shown to promote the enhancement of TRPV4 expression and the release of both IL-1β and TNF-α, which are responsible for infrasound-induced neuronal apoptosis [47]. Furthermore, osmolarity-activated TRPV4 increases the production of IL-1β and IL-6 in intervertebral disc cells [51]; during acute retinal detachment-induced swelling of Müller cells, TRPV4 activation led to MCP-1 release and photoreceptor death [46]. A recent study showed that elevated IOP stimulates TNF-α output through mechanisms potentially dependent on the activation of NMDA receptors, ephrinB/EphB forward signaling, and the precursor form of nerve growth factor signaling on Müller cells [34, 52, 53]. Our study revealed another mechanism dependent on TRPV4 activation, which leads to increased TNF-α production.

There is increasing evidence that the neuroinflammation induced by excessive TNF-α, which is released from activated retinal glial cells, has a critical role in the onset of RGC damage in glaucoma [54]. The evidence suggests that TNF-α-induced RGC death in glaucoma could be mediated by multiple pathways. First, TNF-α could mediate cell death via binding to TNFR1, a death receptor that triggers the extrinsic apoptosis pathway; the expression of TNFR1 in RGCs has been shown to increase after TRPV4 agonist treatment [34, 43, 55, 56]. In a COH model, treatment with soluble TNF-α induced endocytosis of the AMPA receptor GluA2 subunit in RGCs while activating Ca^2+^-permeable GluA2-deficient AMPA receptors in RGCs; these changes promoted RGC death [43]. Another study showed that TNF-α could enhance RGC excitability by upregulating Nav1.6 channels via TNFR1 activation, thus contributing to RGC apoptosis [49].

Importantly, TRPV4 is abundantly expressed on RGCs [18, 33]. TRPV4 regulates the RGC firing rate by regulating Ca^2+^ influx. Moreover, continuous TRPV4 activation causes apoptosis in isolated RGCs [18] and can enhance membrane excitability by reducing Na + current delay time after depolarizing pulses [24]. These results suggest that TRPV4 activation may directly lead to RGC apoptosis through RGC overexcitation. The substantial calcium ion influx induced by TRPV4 activation may also trigger multiple calcium cascades [57] that affect RGC apoptosis in glaucoma; further studies are needed to determine how TRPV4 expression by RGCs directly affects RGC apoptosis. Considering that TRPV4 can mediate RGC apoptosis through diverse mechanisms that involve Müller cells and RGCs, it may serve as a therapeutic target for preventing RGC apoptosis in glaucoma.

### Involvement of JAK2/STAT3/NF-κB signaling pathway in TRPV4 activation-mediated TNF-α production in Müller cells

The mechanism by which TRPV4 mediates Müller cell activation and pro-inflammatory cytokine upregulation is an important research focus. Previous studies demonstrated that the JAK2–STAT3 pathway is involved in glial activation and cytokine expression [58–60]. Moreover, in the context of neurological inflammatory signaling cascades, TRPV4 reportedly exacerbates neuro-inflammatory actions through the activation of pro-inflammatory STAT3 signaling [61, 62]. Furthermore, the inhibition of STAT3 phosphorylation via JAK2 blockade has been shown to reduce hypoxic ischemia-induced neuroinflammation and tissue loss [63]. Consistent with these prior observations, we found that TRPV4 promoted the phosphorylation of JAK2 and STAT3; this effect was enhanced by the application of a TRPV4 agonist. Thus, TRPV4 presumably mediates the activation of Müller cells and the expression of TNF-α via the JAK2/STAT3 pathway.

The NLRP3 inflammasome is a multi-protein complex, in which NLRP3 interacts with the adaptor protein ASC to enable the recruitment and activation of caspase-1, leading to the maturation of IL-1β and IL-18. This complex is responsible for activating the inflammatory response and has important roles in both innate immunity and inflammation-related diseases. Previous studies have shown that Ca^2+^ elevation, reactive oxygen species production, cytosolic potassium depletion, and lysosome disruption can activate this inflammasome [64, 65]. In the present study, the application of a TRPV4 agonist did not increase the expression levels of NLRP3 or caspase-1, indicating that the NLRP3 inflammasome was not involved in TRPV4-induced release of TNF-α.

Activated STAT3 reportedly enables NF-κB dimers to enter the nucleus and bind to specific target genes that mediate the inflammatory response [66]. This notion is supported by our findings. Furthermore, because TRPV4 is Ca^2+^-permeable, its activation could induce excessive Ca^2+^ influx [67]. Previous studies have shown that Ca^2+^ may be involved in JAK2 phosphorylation [68]; thus, we suspect that TRPV4 activation could influence TNF-α release through the Ca^2+^-dependent JAK2/STAT3/NF-κB signaling pathway.

## Conclusions

**Fig. 12:**
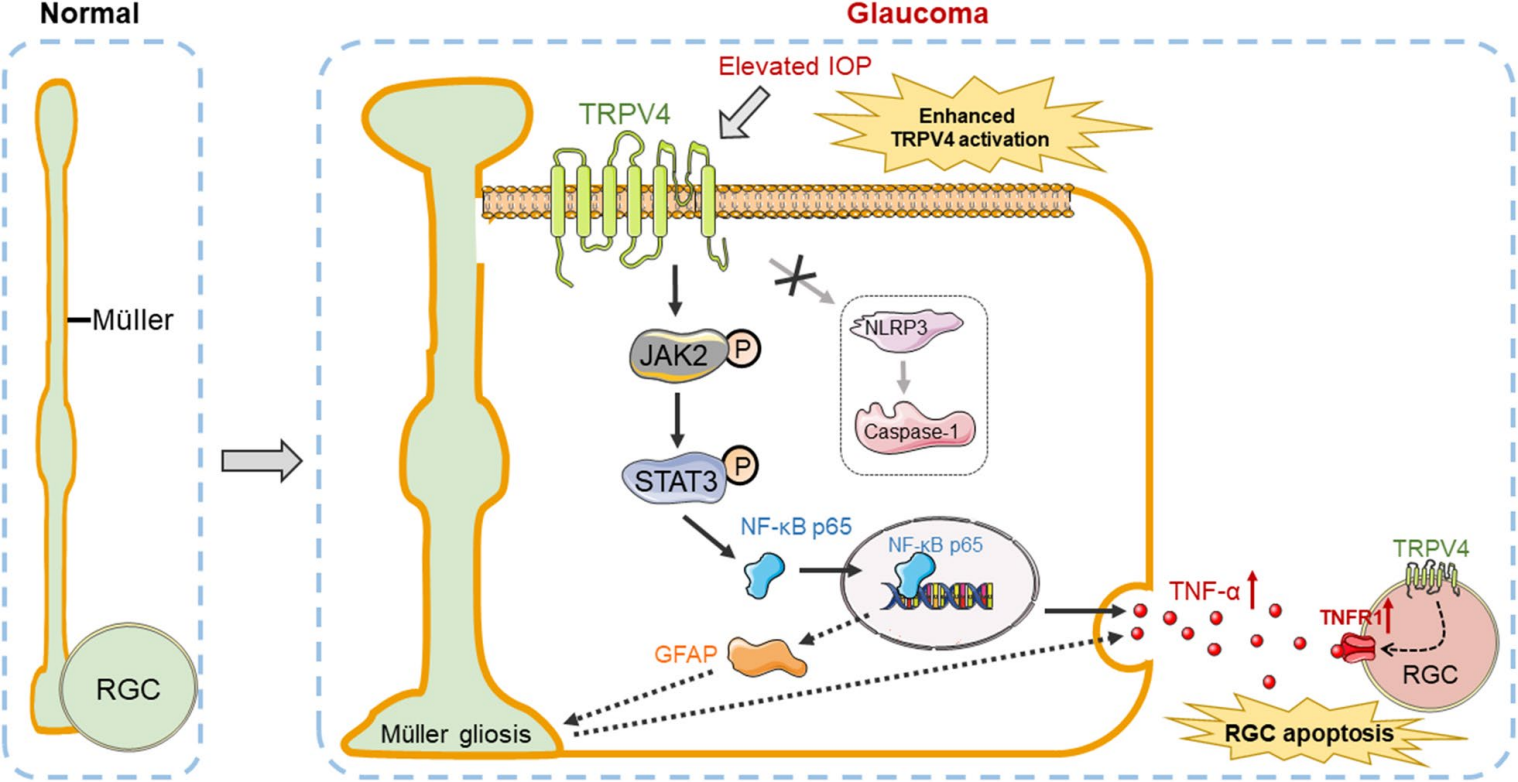
Schematic diagram showing the signaling pathway involved in TRPV4 activation-mediated TNF-α production in Müller cells and RGC apoptosis in COH retinas. *NF-κB* nuclear factor-kappa B, *TNF-α* tumor necrosis factor-α, *TNFR1* TNF receptor 1

Many studies have been conducted concerning potential mechanisms of RGC apoptosis in glaucoma; such mechanisms include cell excitotoxicity, axonal transport disorder, nutrient factor deficiency, and inflammation [3, 4, 69]. However, the factors that directly initiate RGC apoptosis by means of elevated IOP are unknown. We presumed that the mechanosensitive TRPV4 and Piezo channels in the retina could be activated during the onset of glaucoma, thereby triggering multiple subsequent interactions that involve Ca^2+^ influx [70]. Although our findings indicate that elevated IOP can promote enhanced TRPV4 expression in the retina, potentially via TRPV4 activation; it remains unclear whether this is a direct consequence of stress or an indirect effect of another mechanism [17], and additional studies are needed to explore the underlying relationship. Importantly, this activation can directly cause Müller cells to release inflammatory factors; it may also lead to the indirect release of inflammatory factors via Müller cell gliosis, thereby aggravating RGC apoptosis in glaucoma (Fig. 12). Our findings suggest that pre-inhibition of TRPV4 may alleviate pathogenic changes in the expression levels of multiple other proteins related to elevated IOP-induced Ca^2+^ influx; moreover, TRPV4 may serve as a therapeutic target in clinical studies.

## Supplementary Information

**Additional file 1: Fig. S1.** Effects of GSK101 on a-wave and b-wave amplitudes in scotopic ERG. A, Representative scotopic ERG results at 1 week after GSK101 injection. B, Data analyses of a-wave and b-wave amplitudes in scotopic ERG at 1 week after GSK101 injection, *n* = 4, *p < 0.05.

## Data Availability

All data generated or analyzed during this study are included in the published article.

## Abbreviations

COH: Chronic ocular hypertension
GFAP: Glial fibrillary acidic protein
IOP: Intraocular pressure
NF-κB: Nuclear factor-kappa B
PhNR: Photopic negative response
RGC: Retinal ganglion cell
TNF-α: Tumor necrosis factor-α
TNFR1: TNF receptor 1
TRPV4: Transient receptor potential vanilloid 4
TUNEL: Terminal dUTP nick end labeling
