# Supplementary figures and images for: TRPV4-induced Müller cell gliosis and TNF-α elevation-mediated retinal ganglion cell apoptosis in glaucomatous rats via JAK2/STAT3/NF-κB pathway
Source: J Neuroinflammation. 2021 Nov 17;18:271. doi: 10.1186/s12974-021-02315-8 (PMC8596927; doi:10.1186/s12974-021-02315-8)

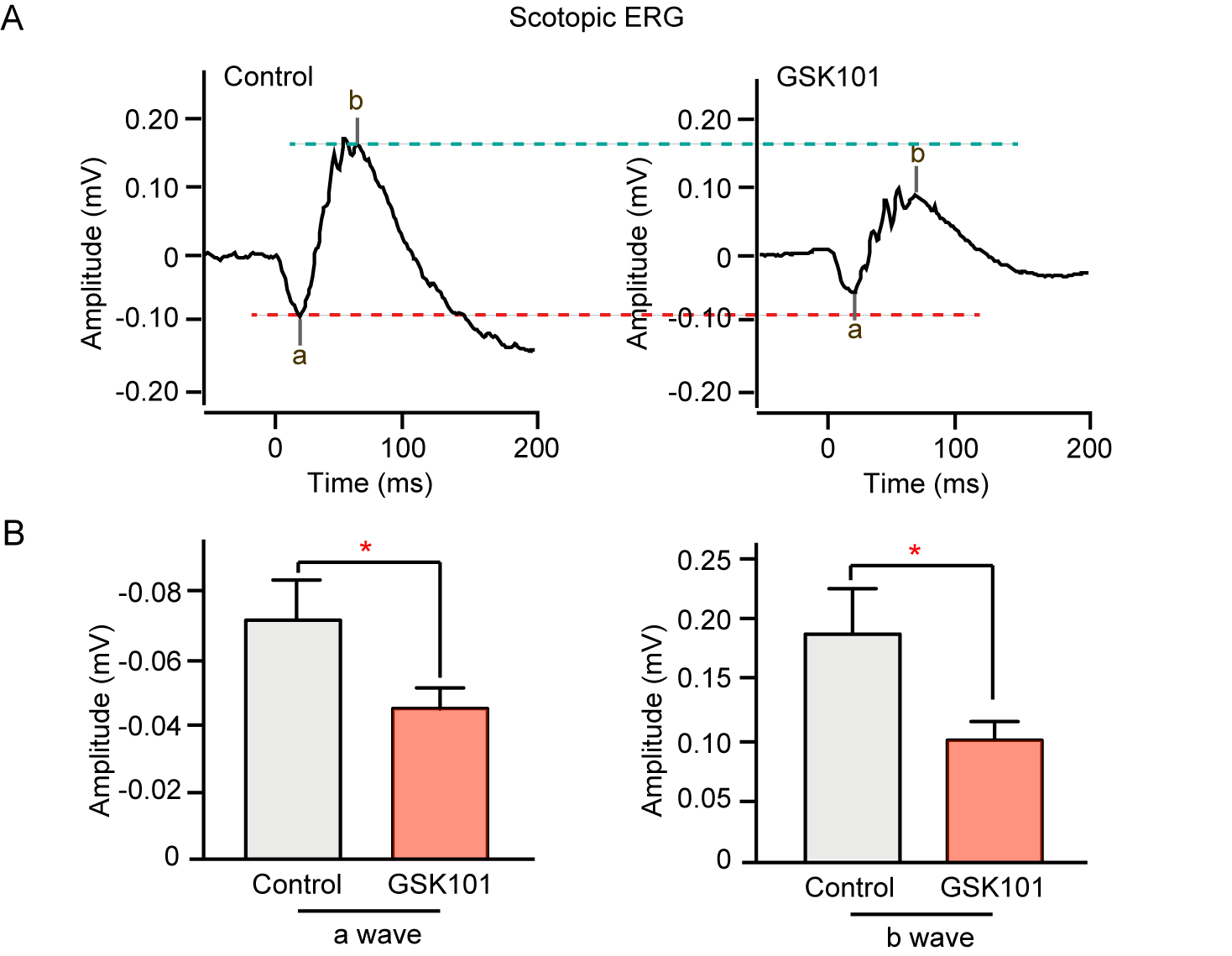

Supplement: Supplementary file 1 — Additional file 1: Fig. S1. Effects of GSK101 on a-wave and b-wave amplitudes in scotopic ERG. A, Representative scotopic ERG results at 1 week after GSK101 injection. B, Data analyses of a-wave and b-wave amplitudes in scotopic ERG at 1 week after GSK101 injection, n = 4, *p < 0.05. [file 12974_2021_2315_MOESM1_ESM.tif]
